# Intracellular expression of arginine deiminase activates the mitochondrial apoptosis pathway by inhibiting cytosolic ferritin and inducing chromatin autophagy

**DOI:** 10.1186/s12885-020-07133-4

**Published:** 2020-07-16

**Authors:** Qingyuan Feng, Xuzhao Bian, Xuan Liu, Ying Wang, Huiting Zhou, Xiaojing Ma, Chunju Quan, Yi Yao, Zhongliang Zheng

**Affiliations:** 1 State Key Laboratory of Virology, College of Life Sciences, Wuhan University, Wuhan, 430072 China; 2 Department of Otorhinolaryngology Head and Neck Surgery, the First Affiliated Hospital of Guangxi Medical University, Nanning, 530021 Guangxi China; 3 Department of Oncology, Renmin Hospital of Wuhan University, Wuhan, 430060 China

**Keywords:** Arginine deprivation, Arginine deiminase, Apoptosis, Mitochondrial damage, Chromatin autophagy

## Abstract

**Background:**

Based on its low toxicity, arginine starvation therapy has the potential to cure malignant tumors that cannot be treated surgically. The Arginine deiminase (ADI) gene has been identified to be an ideal cancer-suppressor gene. ADI expressed in the cytosol displays higher oncolytic efficiency than ADI-PEG20 (Pegylated Arginine Deiminase by PEG 20,000). However, it is still unknown whether cytosolic ADI has the same mechanism of action as ADI-PEG20 or other underlying cellular mechanisms.

**Methods:**

The interactions of ADI with other protein factors were screened by yeast hybrids, and verified by co-immunoprecipitation and immunofluorescent staining. The effect of ADI inhibiting the ferritin light-chain domain (FTL) in mitochondrial damage was evaluated by site-directed mutation and flow cytometry. Control of the mitochondrial apoptosis pathway was analyzed by Western Blotting and real-time PCR experiments. The effect of p53 expression on cancer cells death was assessed by siTP53 transfection. Chromatin autophagy was explored by immunofluorescent staining and Western Blotting.

**Results:**

ADI expressed in the cytosol inhibited the activity of cytosolic ferritin by interacting with FTL. The inactive mutant of ADI still induced apoptosis in certain cell lines of ASS- through mitochondrial damage. Arginine starvation also generated an increase in the expression of p53 and p53AIP1, which aggravated the cellular mitochondrial damage. Chromatin autophagy appeared at a later stage of arginine starvation. DNA damage occurred along with the entire arginine starvation process. Histone 3 (H3) was found in autophagosomes, which implies that cancer cells attempted to utilize the arginine present in histones to survive during arginine starvation.

**Conclusions:**

Mitochondrial damage is the major mechanism of cell death induced by cytosolic ADI. The process of chromatophagy does not only stimulate cancer cells to utilize histone arginine but also speeds up cancer cell death at a later stage of arginine starvation.

## Background

Tumor starvation therapy has become a mainstream strategy for cancer therapy in clinic. In addition to starvation therapy through inhibition of angiogenesis [1], the deprivation of specific amino acids is also a potential cancer therapy. As a potential anti-cancer drug, ADI-PEG20 has already demonstrated some promising results in Phase I and II clinical studies [2, 3]. ADI-PEG20 exhausts the serum arginine thus starving some specific tumors. Those tumors are unable to synthesize arginine due to a deficiency of the enzyme argininosuccinate synthetase (ASS) [4]. David K. Ann and Hsing-Jien Kung [5, 6] et al. described the mechanism through which ADI-PEG20 leads to arginine deprivation in vitro to specifically kill tumor cells, which is actually a novel mechanism involving mitochondrial dysfunction, generation of reactive oxygen species, nuclear DNA leakage, and chromatin autophagy. DNA damage caused by chromatin autophagy triggered the death of cancer cells. However, ADI-PEG20 displayed a lower efficiency in oncolysis. Arginine deprivation in blood only persisted for 2 weeks in an ASS1-methylated malignant pleural mesothelioma [7]. Subsequently, plasma arginine levels recovered due to the development of anti-ADI neutralizing antibodies during the fourth week [7]. ADI-PEG20 monotherapy did not exhibit an overall survival benefit for hepatocellular carcinoma (HCC) patients in Phase III clinical studies [8]. Therefore, new strategies are needed to synergize the effect of ADI-PEG20 in vivo or transform the application methods of the ADI gene in clinical practice.

The ADI gene is a potential cancer suppressor gene [9]. ADI expressed in cytosol displayed a higher apoptosis-inducing efficiency than ADI-PEG20. Cytosolic ADI quickly eliminated cytosolic arginine in the cytoplasm [9] to cause rapid cancer cell death. ADI adenovirus also presented an excellent oncolytic efficiency [9]. Moreover, the promoter of human telomerase reverse transcriptase (hTERT) was utilized to control ADI expression in adenovirus, which ensured higher safety levels for normal cells [9]. Nonetheless, the underlying interaction mechanisms of ADI expressed in the cytosol, or the cellular response to rapid endogenous arginine deprivation are yet to be completely understood. The solution to these issues would effectively prevent side effects when the ADI gene is used for cancer gene therapy in the future.

Here, we aimed to exploit intracellular components that may interact with ADI and figure out whether these interactions are lethal. We sought to identify the molecular determinants of cancer cell death induced by cytosolic ADI, which could serve as a guide for application of the ADI gene in clinic and highlight the choice of agents to be used in combination therapy. We found out that cytosolic ADI interacted with FTL in the cytoplasm and we also detected minor mitochondrial damage. Notwithstanding, arginine deprivation activated the apoptosis pathway of mitochondria control. The increased expression of p53 and p53AIP1 led to mitochondrial damage at the early stage of arginine deprivation. At the later stages of arginine deprivation, chromatin autophagy became worse, which in turn aggravated the mitochondrial damage. Thus, we defined the mechanism underlying the sensitivity of mitochondrial damage to cytosolic ADI and then identified the role of autophagy during arginine deprivation.

## Methods

### Plasmid construction

To construct the pcDNA4-ADI, which is an ADI-overexpressing plasmid, an ADI coding sequence was synthesized using the Nanjing Genscript LTD and then sub-cloned into the *EcoR* I/*Xho* I sites of a pcDNA™4/TO/myc-His vector. The c-myc tag was fused at the c-terminal of the ADI protein. Two primers were used (5′- GATATGAATTCACCATGTCCGTCTTCGAT AGCAAGT − 3′ and 5′- GATATCTCGAG TCACCATTT GACATCTTTTCTGGACA − 3′). The pcDNA4-ADI△(cysteine398alanine) plasmid was created through an overlapping extension method. Two mutant primers were used (5′ GTATGGGTAACG CTCGTGCCATGTCAATGCCTTTATC 3′ and 5′ GATAAAGGCATTGACATGG CACGAGCGTTACCCATAC 3′).

In order to build the pGBKT7-ADI plasmid serving as screening bait through a yeast hybrid experiment, an ADI coding sequence was inserted into the Nde I/BamH I sites of *a* pGBKT7 vector which expresses proteins fused to amino acids 1–147 of the GAL4 DNA binding domain. Two primers were used (5′- GATATCATATGTCCGTCTTCGATAGCAAG TT − 3′ and 5′- GATATCTCGAGTCACCATTT GACATCTTTTCTGGACA − 3′).

Other plasmids were donated by Dr. Youjun Li from the College of Life Sciences at Wuhan University.

### Cell culture and cell lines

Human liver cancer cell lines (HepG2), Prostate cancer cell lines (PC3), and human embryo lung cell lines (MRC5) were cultured with DMEM supplemented with 10% fetal bovine serum (FBS), penicillin (100 IU/ml) and streptomycin (100 μg/ml). Cells were then grown in a 5% CO2 cell culture incubator at 37 °C. All the culture reagents were purchased from Life Technologies LTD. Three cell lines including HepG2 (Cat. #GDC141), PC3 (Cat. #GDC095) and MRC5 (Cat. #GDC032) were purchased from China Center for Type Culture Collection (CCTCC) in July 2017. No mycoplasma contamination was detected in these cells. STR genotypes of three cell lines were tested again in August 2019. The proofs of purchase and the test reports were described in Supplementary information 2.

### Yeast two-hybrid assay

A yeast two-hybrid analysis was performed in *yeast strain AH109* according to the manufacturer’s instructions (http://www.clontech.com/). The pGBKT7-ADI plasmid, used as bait plasmid was co-transformed into the AH109 yeast strain with the yeast two-hybrid cDNA library of the human liver (Cat. #630468) from Clontech Laboratories Inc. A quadruple dropout medium (without tryptophan, leucine, histidine, and adenine) containing 4 mg/ml x-a-gal was used to test the activation of reported genes MEL1 (MDS1/EVI1-like gene 1).

### RNA isolation and quantitative RT-PCR

Total RNA was extracted from the cells using Trizol (Invitrogen) following the manufacturer’s instructions. RNA concentration and purity were both determined by spectrophotometry (NanoDrop Technologies Inc., LLC). One microgram of total RNA was utilized as template for synthesizing complementary DNA strands (cDNA) by using the cDNA Synthesis Kit (Thermo Scientific). Quantitative RT-PCR (qRT-PCR) was performed by using SYBR Green PCR Master Mix with the StepOne Real-Time PCR System (Bio-Rad). 2^-△△Ct^ in the relative quantification analysis method was used to calculate the change fold of mRNA among the different cells. GAPDH was implemented as an internal control for normalization. The primers used for RT-PCR were listed in supplementary Tab S1.

### Western blot analysis

Five micrograms of protein were electrophoresed in 10% SDS-PAGE gels and blotted to polyvinylidene difluoride membranes. Specific primary antibodies were detected with peroxidase-labeled secondary antibodies (ProteinTech Group Inc.) by using SuperSignal West Dura Extended Duration Substrate (Pierce Chemical) per the manufacturer’s instructions. The antibodies used from ProteinTech Group Inc. included the myc-tag antibody (Cat. #66036–1-Ig), ASS antibody (Cat. #66036–1-Ig), GAPDH antibody (Cat. #60004–1-Ig), FTL antibody (Cat. #10727–1-AP), Flag-tag antibody (Cat. # 66008–3-Ig), p53 antibody (Cat. #60283–2-Ig), Bcl-2 antibody (Cat. #60178–1-Ig), PUMA antibody (Cat. # 55120–1-AP), Bax antibody (Cat. #60267–1-Ig), caspase 9 antibody (Cat. # 66169–1-Ig), caspase 3 antibody (Cat. # 66470–2-Ig), Histone H3 antibody (Cat. # 17168–1-AP), HRP-conjugated goat anti-mouse IgG (Cat. #SA00001–1) and HRP-conjugated goat anti-rabbit IgG (Cat. #SA00001–2). The p53AIP1 antibody (Cat. # ABP56144) was supplied by Abbkine Inc., while the Noxa antibody (Cat. # ab13654) and the Bak antibody (Cat. # ab69404) were both from Abcam Inc. The TRITC conjugated goat anti-rabbit antibody (Cat. # AS10–1018) was from Agrisera Inc.

### Fluorescence assay for mitochondrial permeability transition pore (MPTP)

MPTP activation assay followed the manuscript of LIVE Mitochondrial Transition Pore Assay Kit (GMS10095.1 v.A) from GENMED SCIENTIFICS INC. U.S.A. The cells were inoculated in 96-well plates at a density of 5 × 10^4^ cells per well and transfected with the pcDNA4-ADI plasmid. After incubation for 48 h, the cells were then stained with 50 μg calcein-AM (Calcein acetoxymethyl ester), washed with a 0.1 M phosphate buffer solution (PBS), and neutralized with a 0.1 M cobalt (II) chloride hexahydrate. Finally, the cells’ fluorescence intensity was detected in a Thermo Multiskan™ FC Microplate Reader.

### GFP-LC3 reporter fluorescence assay for autophagy in live cells

Expression of the GFP-LC3 fusion gene allows for real-time visualization of autophagosome formation in live cells. Firstly, the cells were inoculated in twelve-well plates with coverslips at a density of 1 × 10^5^ cells per well, and co-transfected with pcDNA4-ADI and pEGFP-LC3 plasmids. Secondly, the cells were starved with in a serum-free medium for 72 h. Thirdly, the cells were fixed with 4% paraformaldehyde, and permeabilized with 0.2% Triton X-100. Cellular nuclei were stained by DAPI for 10 min. Finally, the plates were sealed and stored at 4 °C. GFP fluorescent signals were observed by using a confocal microscope (Leica microsystems, Mannheim, Germany).

### Chromatin autophagy assay by fluorescence co-localization

The cells were inoculated in twelve-well plates with coverslips at a density of 1 × 10^5^ cells per well, and co-transfected with pcDNA4-ADI and pEGFP-LC3 plasmids. Then, 2% FBS was added into the DMEM medium to prevent the cells from dying too quickly. After a culture duration of 96 h, the cells were fixed with 4% paraformaldehyde, and permeated with 0.2% Triton X-100. Afterwards, the cells were incubated with the TRITC-labeled anti-H3 antibody for 4 h at 4 °C. After washing, cellular nuclei were stained by DAPI for 10 min. Eventually, the plates were sealed and stored at 4 °C. Fluorescent signals were detected using a confocal microscope.

### Statistical analysis

Data with error bars are presented as mean ± S.D. The student’s two-tailed t-test was used to determine the *p*-value. Differences were considered statistically significant when the p-value was < 0.05.

## Results

### Cancer cells apoptosis induced by ADI expressed in the cytosol

**Fig. 1:**
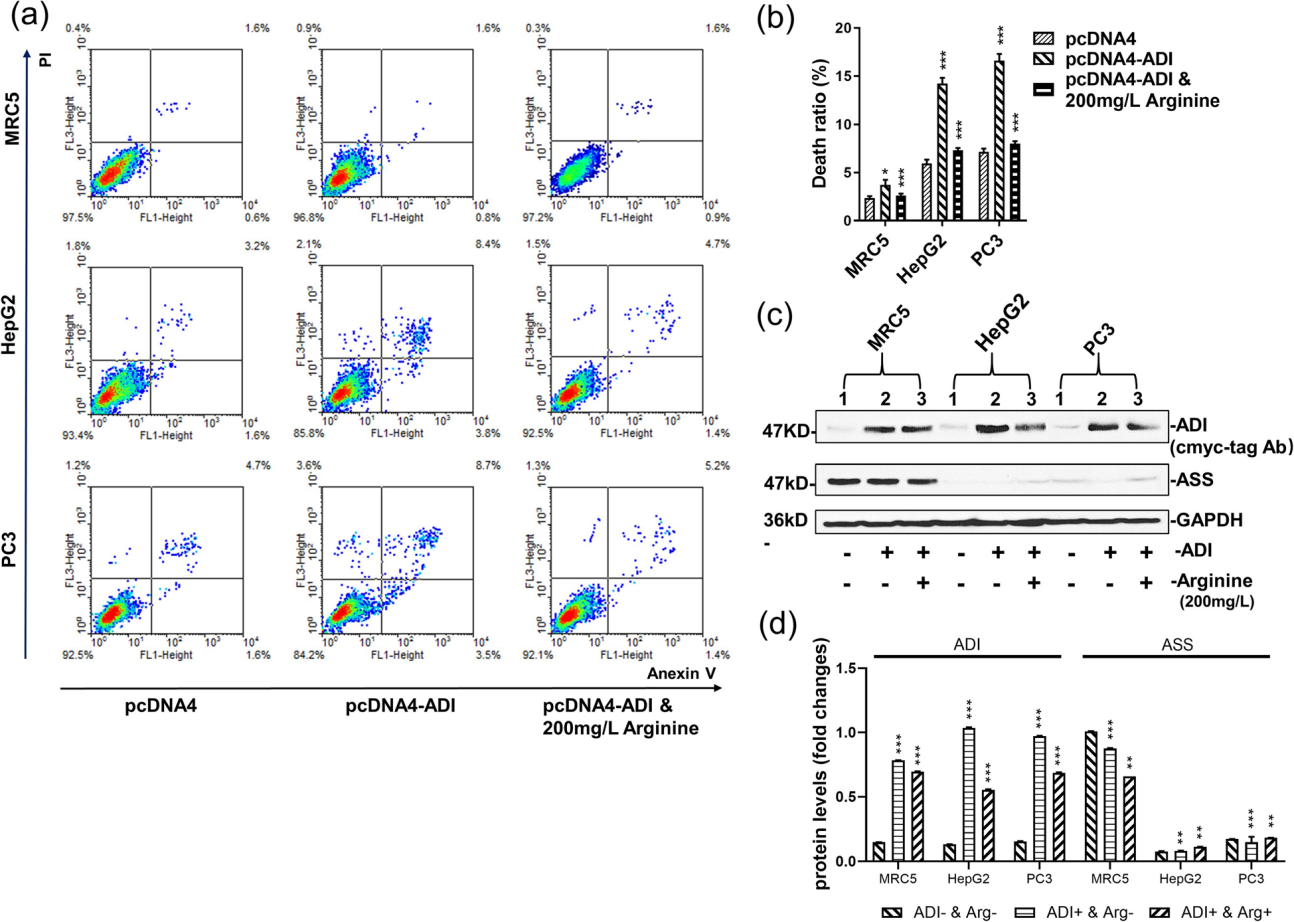
Apoptosis efficiency induced by ADI expressed in MRC5, HepG2 and PC3 cells. Cells were separately transfected by pcDNA4, pcDNA4-ADI plasmids. Cell apoptosis rates were detected by flow cytometry after the static cell culture for 48 h. **a:** Representative images of FACS analysis of annexin V and PI staining of MRC5, HepG2 and PC3 cells. **b:** Death ratio summary of FACS analysis from Fig. 1a. **c:** Immunoblots of ADI and ASS expression in MRC5, HepG2 and PC3 cells. C-myc-tag antibody was used to detect c-myc-tag-fused ADI. The blot of GAPDH was from the same gel as the blot of ADI. Full-length blots are presented in Supplementary Fig. S1. **d**: the relative quantification for protein expressions in MRC5, PC3 and HepG2 cell lines. Grey scales of protein bands from Fig. 1c were detected by ImageJ 1.52. *P* values were calculated by comparing pcDNA4-ADI plasmids-treated cells with pcDNA4 plasmid-treated cells in the respective cell lines. **P < 0.01; ***P < 0.001

ADI expressed in the cytosol was able to efficiently deplete intracellular arginine and lead to cell death. Thus, we transfected the pcDNA4-ADI plasmid into cancer cells to express ADI and determine the apoptosis rate. Based on the cancer tissue specificity of ASS gene expression [4], the MRC5 cell line (ASS+) was used as the negative control, whereas the PC3 (ASS-) and HepG2 (ASS-) cell lines were used as research targets. As indicated by the immunoblotting dots illustrated in Fig. 1c, Fig. 1d and supplementary Fig. S1, the ASS gene was silent in HepG2 and PC3 cells, but highly expressed in MRC5 cells. After 2 days of plasmid transfection, ADI expressed in cytosol efficiently induced the death of the PC3 and HepG2 cells. The apoptosis rate was calculated by summing the rates of early apoptotic cells, late apoptotic cells and dead cells. The PC3 cell line displayed a cell death rate of nearly 17%. The HepG2 cell line also exhibited a cell death rate of roughly 15%. However, ADI demonstrated almost no level of toxicity on normal cells given that the MRC5 cell line experienced a death rate of approximately 4%. 200 mg/L of arginine was used to counteract arginine deprivation induced by cytosolic ADI. The DMEM medium containing 200 mg/L of arginine was replaced every 24 h after transfection of the pcDNA4-ADI plasmid. The high arginine concentration obviously reduced the death rates caused by cytosolic ADI. For example, the HepG2 cells and PC3 cells decreased their death rates to about 7.7 and 8.0%, respectively.

### The interaction between ADI and FTL promoted mitochondrial damage

**Fig. 2:**
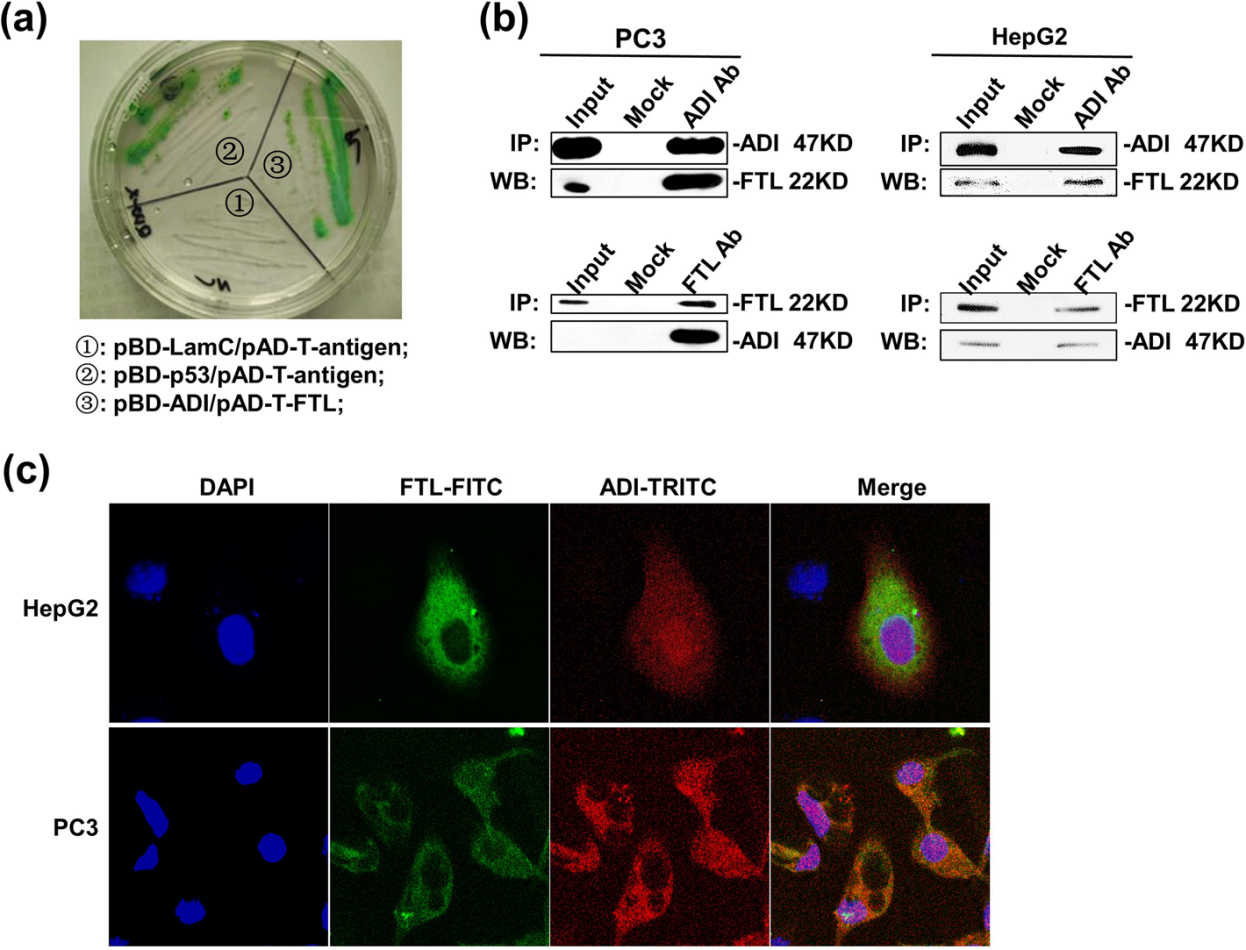
The interaction of ADI and FTL in vivo. **a:** Yeast were co-transformed with pBD-ADI and pAD-T-FTL plasmid, and grew on an SD agar plate with high-stringency nutrient selection (SD/−Leu/−Trp/−His/−Ade). pBD-LamC/pAD-T-antigen plasmids were used as negative control. pBD-p53/pAD-T-antigen plasmids were used as positive control. **b:** Co-IP of ADI or FTL was applied by antibodies specific for ADI or FTL. Images represent the immuneprecipitates separated by SDS-PAGE and incubated with the indicated antibodies. The blots of each line were from the same gel. Full-length blots are presented in Supplementary Fig. S2. **c:** Immunofluorescence staining of HepG2 and PC3 cells with antibody against ADI (red) and antibody against FTL (green). Cells were transfected with pcDNA4-ADI plasmid. The fluorescence was detected on an inverted fluorescence microscope

To understand whether cytosolic ADI has a unique anti-tumor mechanism in cancer cells, we screened several protein factors possibly interacting with ADI by the yeast hybrid method. A cDNA library of human liver from Clontech Laboratories Inc., was used as screening target in the yeast hybrid experiment. As portrayed in Fig. 2a, FTL was screened out and made yeasts display an obvious green color on the selecting plate (SD/Gal/Raf/−Ura, −His, −Trp, −Leu) by interacting with ADI. Next, an immunofluorescence staining was applied to detect intracellular co-localization of ADI and FTL on a confocal microscope. As delineated in Fig. 2c, FTL was located in the cytoplasm and labeled with FITC-green fluorescence. ADI was distributed across the entire cell and labeled with TRITC-red fluorescence. The cytoplasm was clearly their site of interaction as depicted from merged pictures. Co-immunoprecipitation (co-IP) was done to further verify the intracellular interaction between ADI and FTL in ADI-transfected cells. As presented in Fig. 2b and supplementary Fig. S2, FTL was checked out by Western Blotting when ADI was used as IP bait. ADI was also detected by Western Blotting when FTL played the role of IP bait.

**Fig. 3:**
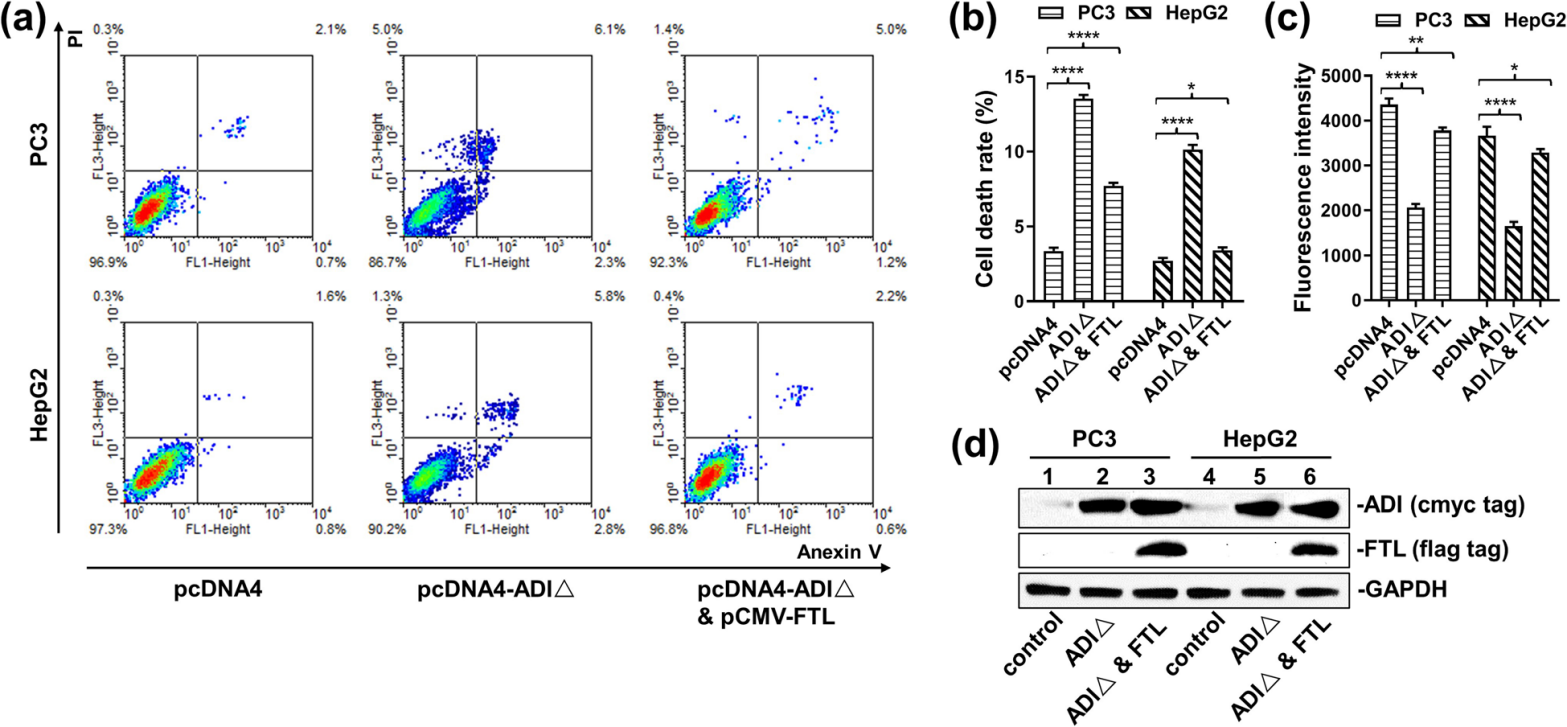
Apoptosis efficiency induced by ADI△(C398A) expressed in MRC5, HepG2 and PC3 cells. Cells were separately transfected by pcDNA4, pcDNA4-ADI△ and pCMV-FTL plasmids. Cell apoptosis rates were detected by flow cytometry after the static cell culture for 72 h. **a:** Representative images of FACS analysis of annexin V and PI staining of MRC5, HepG2 and PC3 cells. **b:** Death ratio summary of FACS analysis from Fig. 3a. **c:** Fluorescence assay for mitochondrial permeability transition pore (MPTP) from Fig. 3a. **d:** Immunoblots of ADI△ and ASS expression in MRC5, HepG2 and PC3 cells. C-myc-tag antibody was used to detect c-myc-tag-fused ADI△. FLAG tag was used to detect overexpressed FTL. The blot of GAPDH was from the same gel as the blot of FTL. Full-length blots are presented in Supplementary Fig. S3

The enzymatic activity of ADI was withdrawn to explore whether ADI could inhibit cytoplasmic FTL through interaction. Considering that the amino acid residue of cysteine398 is the catalytic residue of ADI [10], we mutated cysteine398 into alanine398 to remove the enzymatic activity of ADI. The pcDNA4-ADI△(C398A) plasmid was transfected into PC3 and HepG2 cells to detect cell apoptosis. Subsequently, the pCMV-FTL plasmid was co-transfected to neutralize the action of cytosolic ADI△. As laid out in Fig. 3a, b, d, and supplementary Fig. S3, cytosolic ADI△ still led to 13% of PC3 cell death, and 10% of HepG2 cell death after 3 days of transfection. However, the over-expressed FTL obviously neutralized the death-induced effects in these two cell lines. Co-transfection of the pcDNA4-ADI(C398△A398) and pCMV-FTL plasmids reduced the death rate of PC3 cells to about 7% and HepG2 cells to about 3%. MPTP experiments were further performed to corroborate the mitochondrial damage caused by the cytosolic ADI△. As shown in Fig. 3c, the cytosolic ADI△ decreased half of the fluorescence intensity of the living cells stained by calcein-AM. The co-transfected cells almost kept the same fluorescence intensity as the control cells. Hence, FTL overexpression in vivo prevented mitochondrial damage induced by cytosolic ADI.

### Mitochondria apoptosis pathway induced by arginine deprivation in vivo

**Fig. 4:**
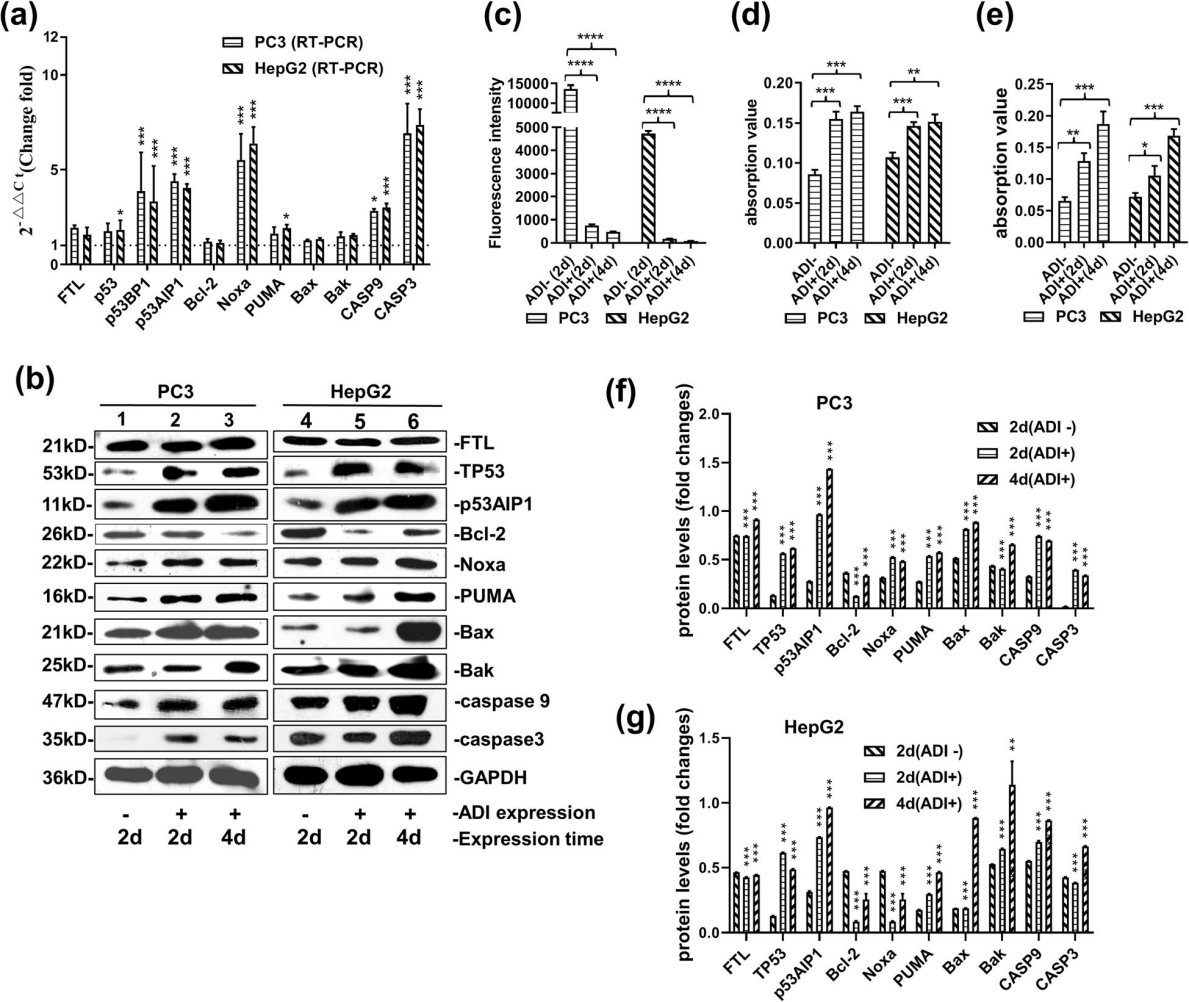
Molecular mechanism of cell apoptosis induced by arginine deprivation. **a:** mRNA level detection of some factors related with mitochondria apoptosis pathway by Quantitative RT-PCR in PC3 and HepG2 cells. **b:** Immunoblot of the factors related with apoptosis pathway in PC3 and HepG2 cells. Full-length blots are presented in Supplementary Fig. S4. **c:** Fluorescence assay for mitochondrial permeability transition pore (MPTP). **d:** Activity assay of Caspase 3 through caspase 3 assay kit (Colorimetric) (*abcam*. ab39401). **e:** Activity assay of Caspase 9 through caspase 9 assay kit (Colorimetric) (*abcam*. Ab65608). **f/g:** The relative quantification for protein expressions in PC3 and HepG2 cell lines. Grey scales of protein bands from Fig. 4b were detected by ImageJ 1.52. *P* values were calculated by comparing pcDNA4-ADI plasmids-treated cells with pcDNA4 plasmid-treated cells in the respective cell lines. **P < 0.01; ***P < 0.001

Mitochondrial apoptosis control pathways were evaluated by fluorescent quantitation RT-PCR and Western Blot experiments. As illustrated in Fig. 4a, after 2 days of ADI expression in cells, the mRNA levels of some important factors increased, such as FTL (about 1.5 fold), p53 (about 1.5 fold), p53AIP1 (about 4.5 fold), Noxa (about 6.0 fold), PUMA (about 1.5 fold), CASP9 (about 3.0 fold) and CASP3 (about 7.0 fold). As shown in Fig. 4b, e, f, and supplementary Fig. S4, the protein levels of these factors also rose after 2 days of arginine deprivation in vivo. Nevertheless, Bax and Bak increased their protein levels on the fourth day of ADI expression. As presented in Fig. 4c, mitochondrial damage was verified by MPTP experiments. The fluorescence intensity of living cells stained by calcein-AM decreased sharply after 2 days or 4 days of arginine deprivation in vivo. As depicted in Fig. 4d and e, the activities of CASP3 and CASP9 simultaneously increased by roughly 1.5 to 2.0 fold.

**Fig. 5:**
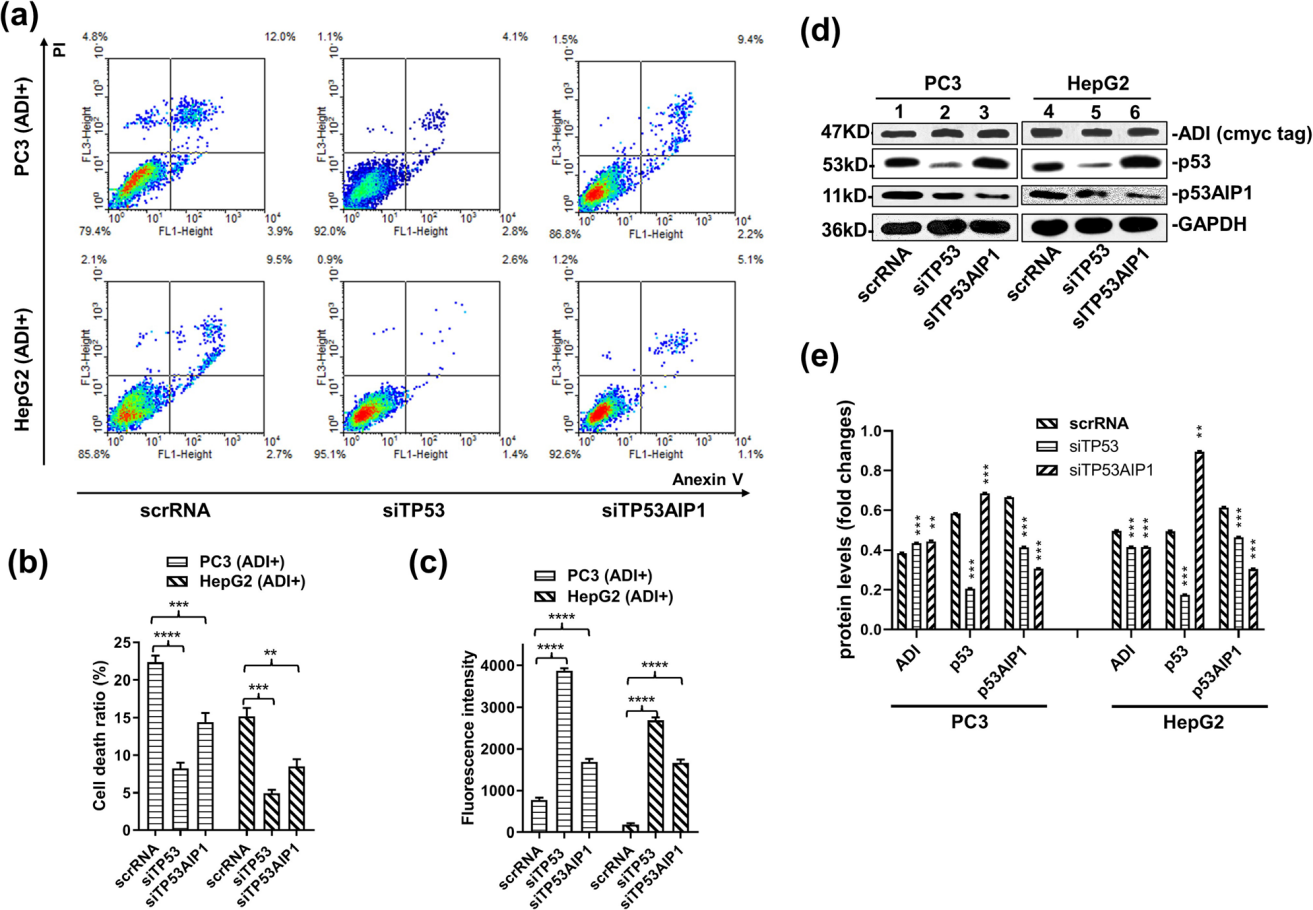
The effect of knock-down of p53 and p53AIP1 genes on apoptosis efficiency induced by ADI. Cells were separately co-transfected by pcDNA4-ADI plasmids with siTP53 or siTP53AIP1. Cell apoptosis rates were detected by flow cytometry after the static cell culture for 48 h. **a:** Representative images of FACS analysis of annexin V and PI staining of HepG2 and PC3 cells. **b:** Death ratio summary of FACS analysis from Fig. 5a. **c:** Fluorescence assay for mitochondrial permeability transition pore (MPTP) from Fig. 3a. **d:** Immunoblots of ADI, p53 and p53AIP1 protein expression in HepG2 and PC3 cells. C-myc-tag antibody was used to detect c-myc-tag-fused ADI. Full-length blots are presented in Supplementary Fig. S5. **e:** the relative quantification for protein expressions in PC3 and HepG2 cell lines. Grey scales of protein bands from Fig. 5d were detected by ImageJ 1.52. *P* values were calculated by comparing siRNA-treated cells with scrRNA-treated cells in the respective cell lines. **P < 0.01; ***P < 0.001

Furthermore, increased levels of p53AIP1 expression activated p53-dependent apoptosis [11]. As a result, we respectively knocked down p53 mRNA and p53AIP1 mRNA to verify their functions in mitochondrial damage during arginine deprivation in vivo. As observed in Fig. 5d and e, the protein levels of p53 and p53AIP1 decreased in the PC3 and HepG2 cell lines after 2 days of arginine deprivation in vivo and siRNA transfection. Knock-down of the p53 mRNA level effectively decreased cell death rates, as displayed by the flow cytometry results in Fig. 5a and b. siTP53AIP1 also reduced cell death rates in PC3 and HepG2 cells. MPTP experiments yielded the same results, revealed in Fig. 5c. The fluorescence intensity of living cells stained by calcein-AM was much higher in siRNA-treated cells than in scrRNA-treated cells.

### Cellular autophagy induced by ADI expressed in the cytosol

Cellular autophagy was detected because nutrient starvation is the major reason to trigger excessive autophagy [12]. Assay for microtubule-associated protein 1A/1B-light chain 3 (LC3) is the basic protocol for the detection of autophagosomes. A cytosolic form of LC3 (LC3-I) is conjugated to phosphatidylethanolamine to generate an LC3-phosphatidylethanolamine conjugate (LC3-II) during autophagy, which is recruited in autophagosomal membranes. Thus, an assay for the formation of GFP-LC3-II can reliably reflect the starvation-induced autophagic activity [13].

**Fig. 6:**
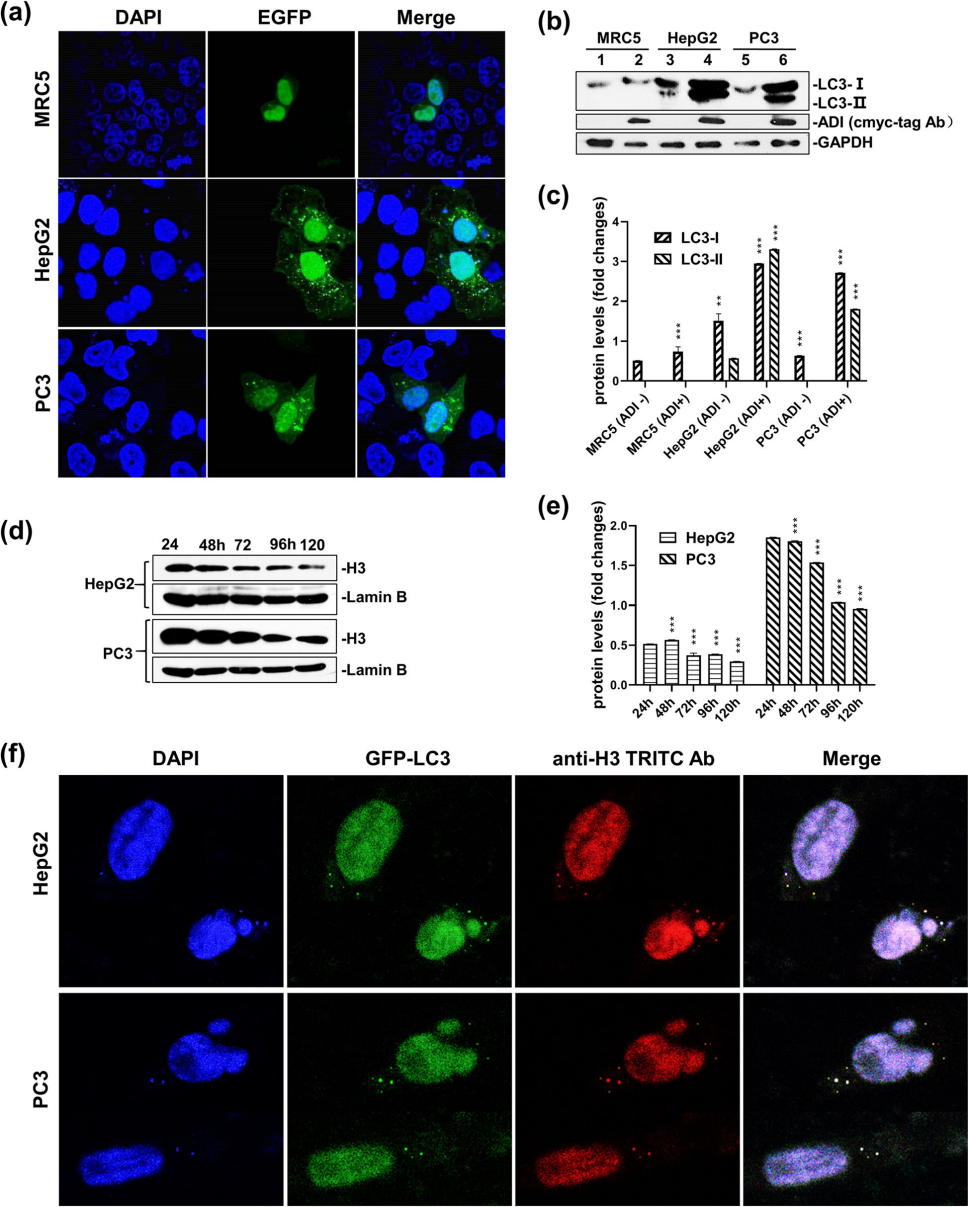
Chromatin autophagy assay at the later time point of arginine deprivation. **a:** GFP-LC3 reporter fluorescence assay for autophagy in MRC5, HepG2 and PC3 cells. Cells were co-transfected with pcDNA4-ADI plasmid and pEGFP-LC3 plasmid. The fluorescence of EGFP protein was detected by OLIMPUS inverted fluorescence microscope SteREO Discovery V12. **b:** Immunoblot of LC3-I and LC3-II in MRC5, HepG2 and PC3 cells. Cells were treated as the description of Fig. 6a. LC3 antibody was used to detect LC3-I and LC3-II proteins. C-myc-tag antibody was used to detect c-myc-tag-fused ADI. Full-length blots are presented in Supplementary Fig. S6A. **c:** the relative quantification for protein expressions in MRC5, PC3 and HepG2 cell lines. Grey scales of protein bands from Fig. 6b were detected by ImageJ 1.52. *P* values were calculated by comparing pcDNA4-ADI plasmids-treated cells with pcDNA4 plasmid-treated cells in the respective cell lines. **P < 0.01; ***P < 0.001. **d:** Immunoblots of H3 protein expression in HepG2 and PC3 cells. Cells were transfected with pcDNA4-ADI plasmid. Histone H3 antibody (Cat. # 17168–1-AP) were used to detect H3 protein. Full-length blots are presented in Supplementary Fig. S6B. **e:** the relative quantification for protein expressions in MRC5, PC3 and HepG2 cell lines. Grey scales of protein bands from Fig. 6d were detected by ImageJ 1.52. *P* values were calculated by comparing other cells with 24 h-treated cells in the respective cell lines. **P < 0.01; ***P < 0.001. **f:** Immunofluorescence assay for chromatin autophagy. Cells were cultured in DMEM medium with 2% FBS. Histone H3 antibody (Cat. # 17168–1-AP) were used to detect H3 protein in cells. TRITC conjugated goat anti-rabbit antibody (Cat. # AS10–1018) was used to detect H3 antibody and display the immunofluorescence

The pcDNA4-ADI and pEGFP-LC3 plasmids were co-transfected into MRC5, PC3, and HepG2 cell lines. After 96 h of co-transfection, GFP fluorescence was detected using a confocal microscope. The protein levels of LC3 were directly verified by Western Blotting. As highlighted in Fig. 6b, c, and supplementary Fig. S6A, LC3-II proteins were only checked out in HepG2 and PC3 cells that expressed ADI proteins. Autophagosomes also appeared in the cytoplasm of the same starved cells as shown in Fig. 6a. Withal, the MRC5 cells did not present any autophagosomes during starvation. At the same time, the protein expression of histone 3 (H3) was inspected by Western Blotting. H3 protein levels decreased hardly after 96 h of arginine deprivation in cells as shown in Fig. 6d, e, and supplementary Fig. S6B.

Chromatin autophagy was further detected through fluorescence co-localization technology. As shown in Fig. 6f, the cell nuclei depicted the budding phenomenon in HepG2 and PC3 cells. There were some autophagosomes appearing in the cytoplasm. The merged pictures revealed that DNA fragments, GFP-LC3-II and histone H3 were located in the same autophagosomes.

## Discussion

Tumors tend to adapt to the microenvironmental changes when they are threatened by death. In clinical practice, some tumors remain in quiescent conditions due to hypoplasia of their supplying blood vessels. Meanwhile, some tumor tissues remain dystrophic since they cannot obtain enough nutrients from hypoplastic blood vessels. Besides, selectively starving cancer cells can also make tumor cells to be malnourished, which is a metabolic-based therapy for cancers with tiny side effects. Cancer-starving therapies, such as dietary modification, inhibition of tumor angiogenesis, and aspartic acid deficiency, can effectively decrease the incidence of spontaneous tumors and slow the growth of primary tumors [14].

ADI is a suitable gene to be targeted for cancer gene therapy. As a description of our preliminary work [9], cytosolic ADI expression displayed a higher apoptosis-inducing efficiency, tumor-targeting specificity, and oncolytic activity [9]. In order to exclude the actions of adenovirus on cells, we just used a pcDNATM4/TO/myc-His vector as an ADI expression vector without replacing the pCMV promoter with a phTERT promoter. The rapid growth of tumors requires a tremendous supply of nutrients including arginine. Tumor cells exhibiting ASS gene deficiency such as endometrial cancer are more sensitive to arginine deprivation than normal cells [15]. Based on the cancer tissue specificity of ASS expression [4], we used MRC5 (ASS+), PC3 (ASS-), and HepG2 (ASS-) cell lines to explore whether ADI had the same effect on different cancer cell lines. As illustrated in Fig. 1, ADI expressed in the cytosol eventually induced cellular apoptosis of PC3 and HepG2 cells.

ADI-PEG20 has been proved to induce cellular autophagy and caspase-independent apoptosis by exhausting the arginine in the peripheral microenvironment of tumors [16]. Notwithstanding, it is unknown whether cytosolic ADI has the same anti-tumor mechanism. We aimed at understanding whether ADI has a unique anti-tumor mechanism in vivo. Consequently, we screened the protein factors that would interact with ADI using the yeast hybrid method. FTL was screened out as revealed in Fig. 2. Co-IP results confirmed the interaction between ADI and FTL in cells. Fluorescence co-localization demonstrated that the interaction happened in the cytoplasm.

Ferritin is considered as the major iron storage protein, which participates in the regulation of cellular iron homeostasis [17]. Mitochondrial function also requires iron replenishment from cytoplasmic ferritin. Thus, inhibition of ferritin directly results in dysfunction of the mitochondrial electron transport chain [18]. To exclude the effect of ADI’s enzymatic activity on cellular metabolism, the catalytic residues of ADI were mutated into alanine residues. Cysteine398, the catalytic residue of ADI [10], was mutated into alanine398. Since alamine398 as an inert residue has no nucleophilic catalytic capacity, the mutation (C398△A398) effectively terminated the enzymatic activity of ADI [19]. As presented in Fig. 3, ADI△(C398△A398) still induced a small number of cell death in PC3 and HepG2 cells. Overexpression of FTL neutralized the apoptotic effects on these two cells. Based on these facts, we speculated that FTL overexpression constituted the part of cytosolic FTL that had lost its function due to interaction with ADI. That said, ADI△(C398△A398) needs 3 days to induce cancer cell death, while ADI only needs 2 days as pointed out in Fig. 2. It can be seen that cytosolic ADI△ just induces a limited level of apoptosis through interacting with cytosolic FTL. The interaction between ADI and FTL is not the main reason for mitochondrial damage. In addition, as represented in Fig. 1, high concentration of arginine in the culture medium counteracted the cell death caused by cytosolic ADI expression. This result further suggests that arginine deprivation in the cytosol is the predominant mechanism for cytosolic ADI suppressing the growth of cancer cells.

Collected pieces of evidence in research papers have proven that arginine deprivation in vitro exerts its anticancer effects on various tumors by inducing mitochondrial damage and autophagy [5, 6, 20, 21]. Additionally, arginine deprivation inhibits nitric oxide synthesis in cells [22, 23]. Thus, arginine deprivation cannot damage the mitochondria by increasing nitric oxide biosynthesis in cells. David K. Ann and Hsing-Jien Kung [24] also reported that mitochondrial damage is the principal explanation for cancer cell apoptosis induced by ADI-PEG20. Our MPTP experiments also confirmed that cytosolic ADI led to serious mitochondrial damage as presented in Fig. 4c. However, the exact mechanism regarding the apoptosis pathway induced by mitochondrial damage during arginine deprivation in vivo is still not clear.

Next, we checked the expression of some protein factors associated to the mitochondrial apoptosis pathway. As demonstrated in Fig. 4a and b, 2 days of arginine deprivation in vivo increased the expression of p53 and p53AIP1 proteins in PC3 and HepG2 cells. Ectopic expression of the p53AIP1 protein induced down-regulation of the mitochondrial Δψ_m_ (transmembrane potential) and release of cytochrome c from the mitochondria by interacting and inhibiting Bcl-2 in the outer membrane of the mitochondria [25]. Clearly, after 2 days of starvation, increase in the expression of the p53AIP1 protein activated p53-dependent apoptosis by interacting with the same upregulated expression of the p53 protein [11, 26]. Ultimately, cytochrome C was released from the mitochondria. Casp3 and Casp9 were activated as delineated in Fig. 4d and e. At the latest stage of arginine deprivation in cells (for 4 days), the PC3 and HepG2 cells seemed to enter the initiative apoptosis process, due to the fact that increasing expression of Noxa, PUMA, Bax and Bak proteins would further aggravate mitochondrial damage [27, 28] as shown in Fig. 4a and b.

We further knocked down the mRNA levels of p53 and p53AIP1 to verify their action during arginine deprivation in cells. As portrayed in Fig. 5a, b, d, and supplementary Fig. S5, the knockdown effectively reduced the apoptosis rates in PC3 and HepG2 cells. p53 knockdown displayed better effects in terms of apoptosis inhibition compared to the p53AIP1 knockdown. Mitochondrial damage was also prevented by p53 knockdown, due to the higher fluorescence intensity of living cells exhibited in Fig. 5c. Consequently, p53-dependent apoptosis pathway was the major pathway induced by cytosolic ADI.

It is worth mentioning that mitochondrial damage was not the only factor leading to cancer cell death during arginine deprivation in the cytosol. Cellular autophagy was also reported to be induced by ADI-PEG20 [16]. Autophagy, the process of cellular self-eating, is usually triggered by starvation or stress, which is capable to degrade long-lived proteins and organelles such as the endoplasmic reticulum, mitochondria, peroxisomes, ribosomes and the nucleus [29, 30]. We also proved that autophagy was induced by cytosolic ADI. The pEGFP-LC3 and pcDNA4-ADI plasmids were co-transfected into cells. With the expression of ADI, more proteins were converted from LC3-I to LC3-II as laid out in Fig. 6b. Cytosolic GFP-LC3-I was conjugated to phosphatidylethanolamine to form GFP-LC3-II during autophagy. GFP-LC3-II was subsequently recruited into autophagosomal membranes during the formation of autophagosomes [31]. As depicted in Fig. 6a, green GFP fluorescent particles presenting around the nucleus were autophagosomes in two cancer cells. Thus, with the expression of ADI, the autophagy induced by arginine starvation was indeed taking place in these cells.

Hsing-Jien Kung reported that arginine deprivation in vitro could lead to cancer cell chromatin autophagy [32]. He equally stipulated that prolonged arginine deprivation would cause mitochondrial dysfunction and generation of ROS, eventually resulting in DNA damage and nuclear membrane remodeling. Excessive autophagy leads to a giant aggregate of autophagosomes/autolysosomes fusion in the late stage of arginine deprivation in vitro. Stephen Gregory [33] disclosed that chromatophagy was necessary for the survival of chromosomal instability in (CIN) cells. Chromatophagy is activated to remove the defective mitochondria in response to DNA damage. However, we had an additional view of chromatophagy. We reckon that arginine deprivation mobilizes cells to utilize endogenous arginine storage. Nucleosomes, especially histone 3 (H3), contain abundant arginine residues. Consequently, the cells attempt to obtain arginine from chromatophagy to maintain basic physiology during arginine deprivation. As displayed in Fig. 6f, nucleus budding occurred in HepG2 and PC3 cells 96 h after co-transfection of the pcDNA4-ADI and pEGFP-LC3 plasmids. Chromatin fragment (blue fluorescence) and H3 proteins (red fluorescence) were displayed to co-localize in autophagosomes (GFP green fluorescence). This showed that ADI expressed in the cytosol also induced chromatin autophagy. H3 proteins present in autophagosomes implied the utility of histones arginine.

## Conclusion

Based on the above discussion, we can see that the death of cancer cells is primarily induced by rapid intracellular arginine deprivation secondary to expression of the ADI gene in the cytosol. Mitochondrial damage is the main pathway of cellular death induced by cytosolic ADI as illustrated in Figure S7. Cytosolic ADI can interrupt the activity of the mitochondrial electron transport chain by interacting with cytosolic FTL. The interaction between cytosolic ADI and FTL only accelerates mitochondrial damage. DNA damage was demonstrated as the major reason for mitochondrial damage. Cytosolic ADI leads to rapid deprivation of cytosolic arginine, which stimulate cancer cells to utilize endogenous sources of arginine. Consequently, the cancer cells initiate chromatin autophagy so as to use the abundant levels of arginine existing in nucleosomes. During the early stage of arginine deprivation in vivo, chromatin autophagy is negligible, but DNA damage induces the increased expression of p53 and p53AIP1 proteins. Subsequently, the interaction between p53 and p53AIP1 further aggravates mitochondrial damage. During the later stage of arginine deprivation in vivo, the rise in chromatin autophagy worsens the DNA damage, which leads to the increased expression of Noxa, PUMA, Bax, and Bak proteins. At this point, mitochondrial damage is far beyond repair, leading to apoptosis (programmed cell death) of the cancer cells. Even though our conclusion is still full of unknowns, we plan to provide a comprehensive explanation of the molecular mechanism regarding the role of arginine deprivation in the activation of chromatin autophagy in the future.

## Supplementary information

**Additional file 1: Figure S1** is associated with Fig. 1c.

**Additional file 2: Figure S2.** is associated with Fig. 2b.

**Additional file 3: Figure S3.** is associated with Fig. 3d.

**Additional file 4: Figure S4.** is associated with Fig. 4b.

**Additional file 5: Figure S5.** is associated with Fig. 5d.

**Additional file 6: Figure S6**A. is associated with Fig. 6b.

**Additional file 7: Figure S6**B. is associated with Fig. 6d. **Figure S7** is mitochondrial apoptosis pathway induced by cytosolic ADI.

**Additional file 8: Table S1.** displays some primers for qPCR experiments.

**Additional file 9.** Cell STR gene type test reports and cell purchase certificate

## Data Availability

All data generated or analyzed during this study are included in this published article [and its supplementary information files]. The gene sequences for plasmid construction are all from NCBI. Accession number of ADI gene is ‘GenBank: X54141.1’ (https://urldefense.proofpoint.com/v2/url?u=https-3A__www.ncbi.nlm.nih.gov_nuccore_X54141.1_&d=DwIGaQ&c=vh6FgFnduejNhPPD0fl_yRaSfZy8CWbWnIf4XJhSqx8&r=Z3BY_DFGt24T_Oe13xHJ2wIDudwzO_8VrOFSUQlQ_zsz-DGcYuoJS3jWWxMQECLm&m=4qSIQc8s5i3dtCx-B-SQ8v47LEypiHbJHd_ZSDQ3qsA&s=txP9mFvMjiOiWgMIID8iL2sijVDKem88fvhgbvuPcmw&e=). Accession number of p53 gene is ‘GenBank: JQ694050.1’ (https://urldefense.proofpoint.com/v2/url?u=https-3A__www.ncbi.nlm.nih.gov_nuccore_JQ694050.1&d=DwIGaQ&c=vh6FgFnduejNhPPD0fl_yRaSfZy8CWbWnIf4XJhSqx8&r=Z3BY_DFGt24T_Oe13xHJ2wIDudwzO_8VrOFSUQlQ_zsz-DGcYuoJS3jWWxMQECLm&m=4qSIQc8s5i3dtCx-B-SQ8v47LEypiHbJHd_ZSDQ3qsA&s=9AY8CMN-ZcJNclmIec4A9szS1JsVtbJmkGubKPb4yDA&e=). Accession number of FTL gene is ‘GenBank: NM_000146.4’ (https://urldefense.proofpoint.com/v2/url?u=https-3A__www.ncbi.nlm.nih.gov_nuccore_NM-5F000146.4&d=DwIGaQ&c=vh6FgFnduejNhPPD0fl_yRaSfZy8CWbWnIf4XJhSqx8&r=Z3BY_DFGt24T_Oe13xHJ2wIDudwzO_8VrOFSUQlQ_zsz-DGcYuoJS3jWWxMQECLm&m=4qSIQc8s5i3dtCx-B-SQ8v47LEypiHbJHd_ZSDQ3qsA&s=fU3MQSzjGMGnAEkTI5UZXcvCaVd9qqiQ6VK7FuFq5fw&e=).

## Abbreviations

ADI: Arginine deiminase
ADI-PEG20: Pegylated Arginine Deiminase by PEG 20,000
FTL: Ferritin light-chain domain
H3: Histone 3
ASS: Argininosuccinate synthetase
HCC: Hepatocellular carcinoma
hTERT: Human telomerase reverse transcriptase
cDNA: Complementary DNA
qRT-PCR: Quantitative reverse transcription-polymerase chain reaction
co-IP: Co-immunoprecipitation
LC3: Microtubule-associated protein 1A/1B-light chain 3
LC3-II: LC3-phosphatidylethanolamine conjugate
CIN cells: Chromosomal instability cells
