# Supplementary figures and images for: Intracellular expression of arginine deiminase activates the mitochondrial apoptosis pathway by inhibiting cytosolic ferritin and inducing chromatin autophagy
Source: BMC Cancer. 2020 Jul 16;20:665. doi: 10.1186/s12885-020-07133-4 (PMC7367323; doi:10.1186/s12885-020-07133-4)

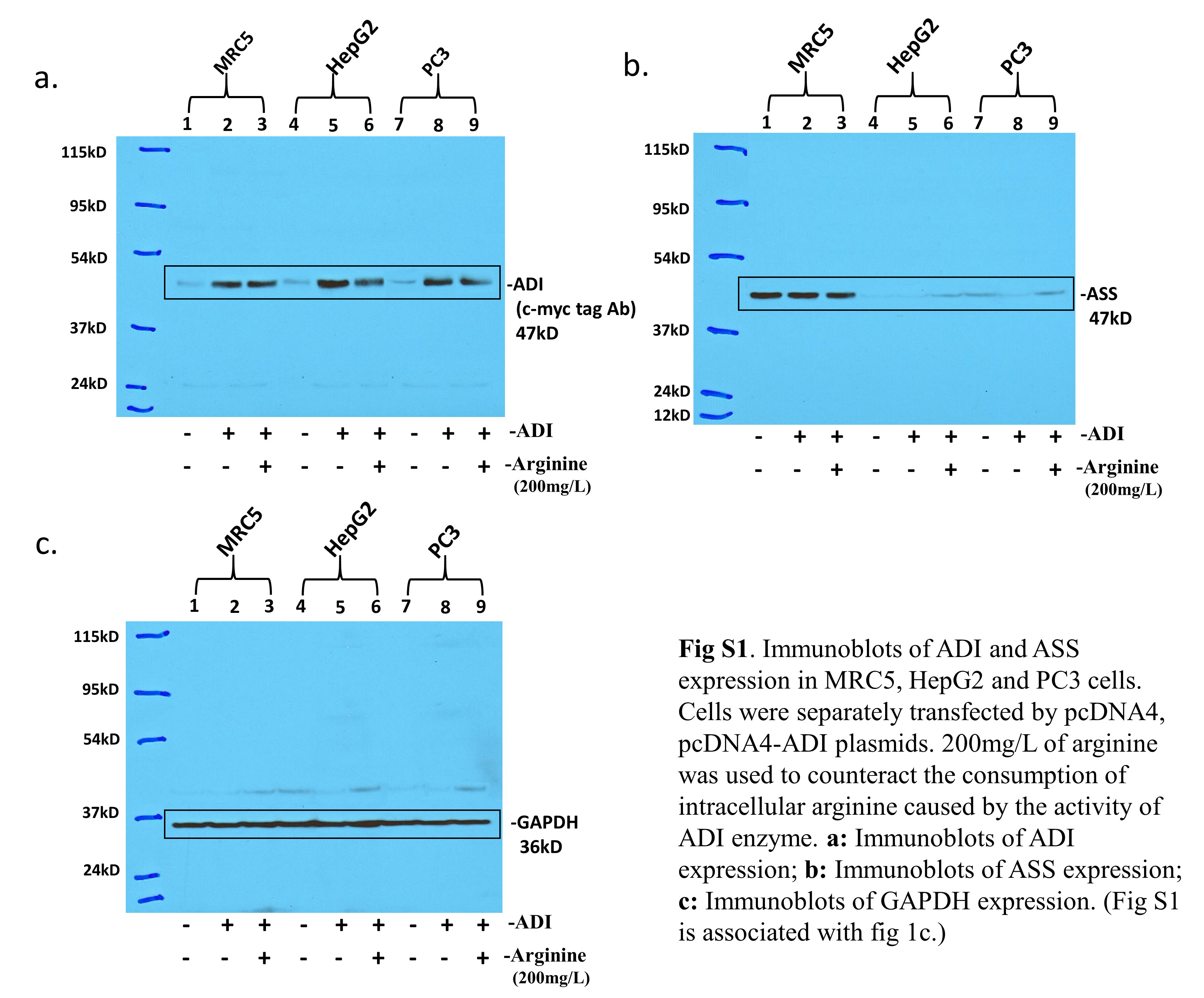

Supplement: Supplementary file 1 — Additional file 1: Figure S1 is associated with Fig. 1c. [file 12885_2020_7133_MOESM1_ESM.jpg]

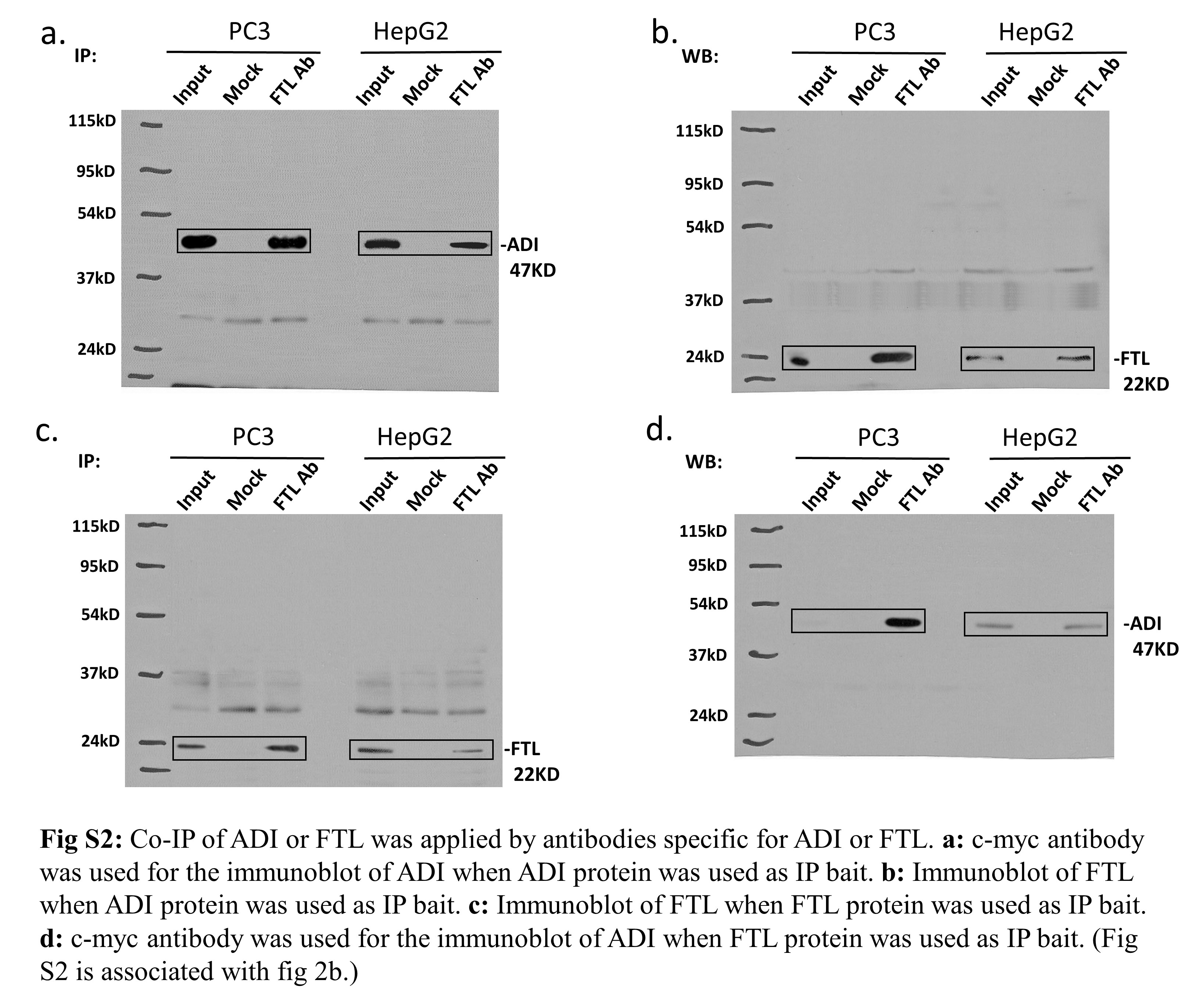

Supplement: Supplementary file 2 — Additional file 2: Figure S2. is associated with Fig. 2b. [file 12885_2020_7133_MOESM2_ESM.jpg]

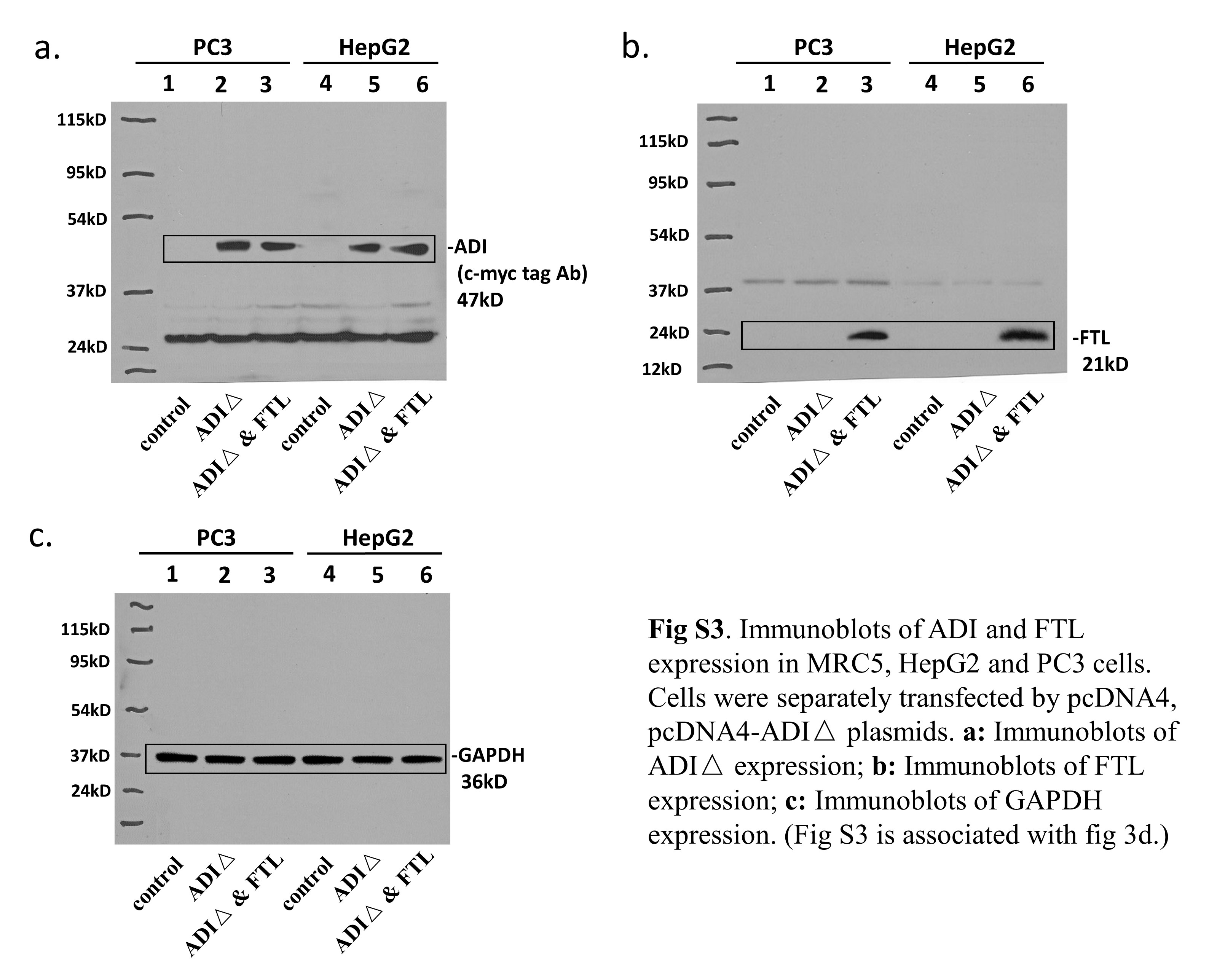

Supplement: Supplementary file 3 — Additional file 3: Figure S3. is associated with Fig. 3d. [file 12885_2020_7133_MOESM3_ESM.jpg]

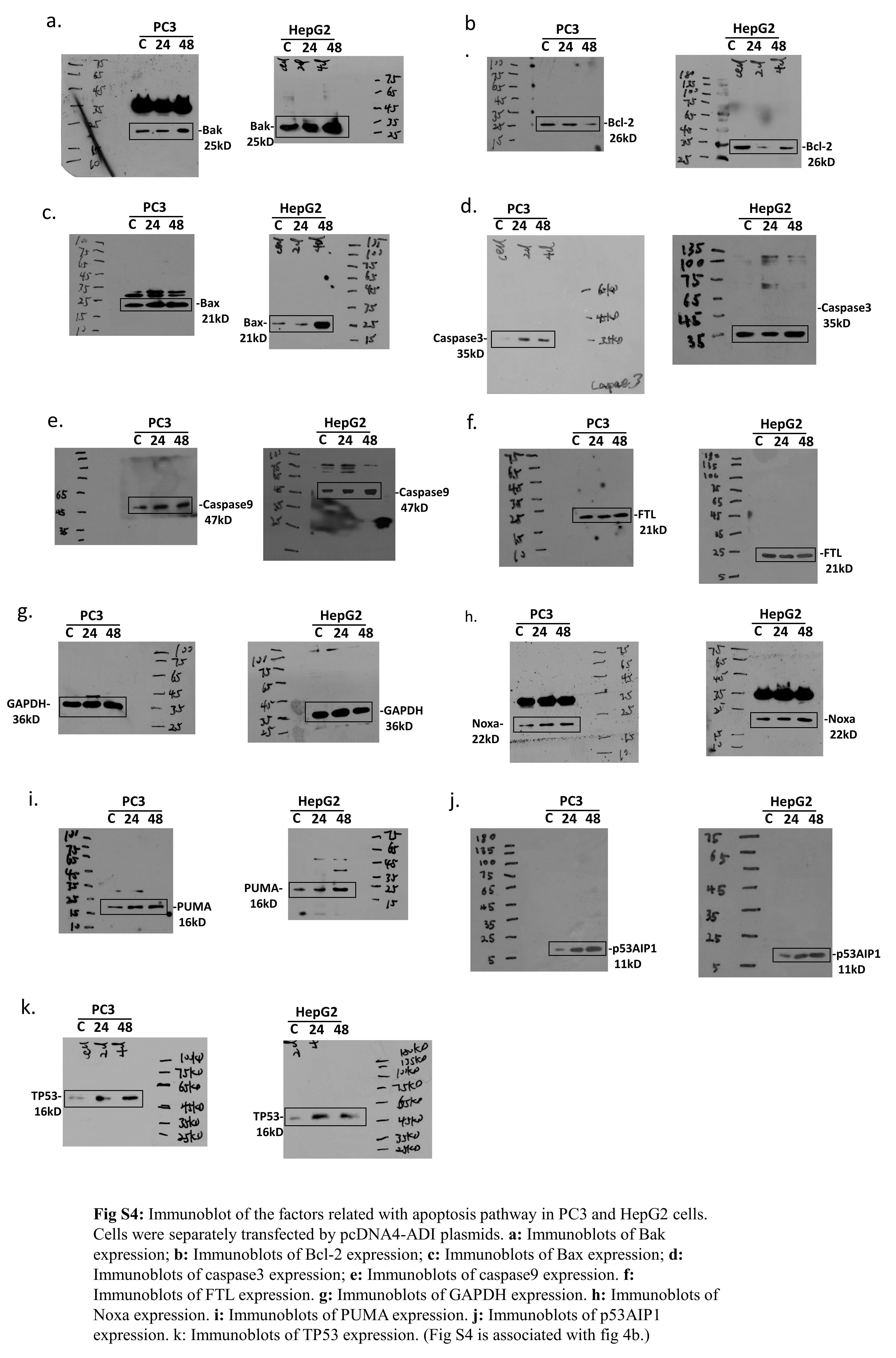

Supplement: Supplementary file 4 — Additional file 4: Figure S4. is associated with Fig. 4b. [file 12885_2020_7133_MOESM4_ESM.jpg]

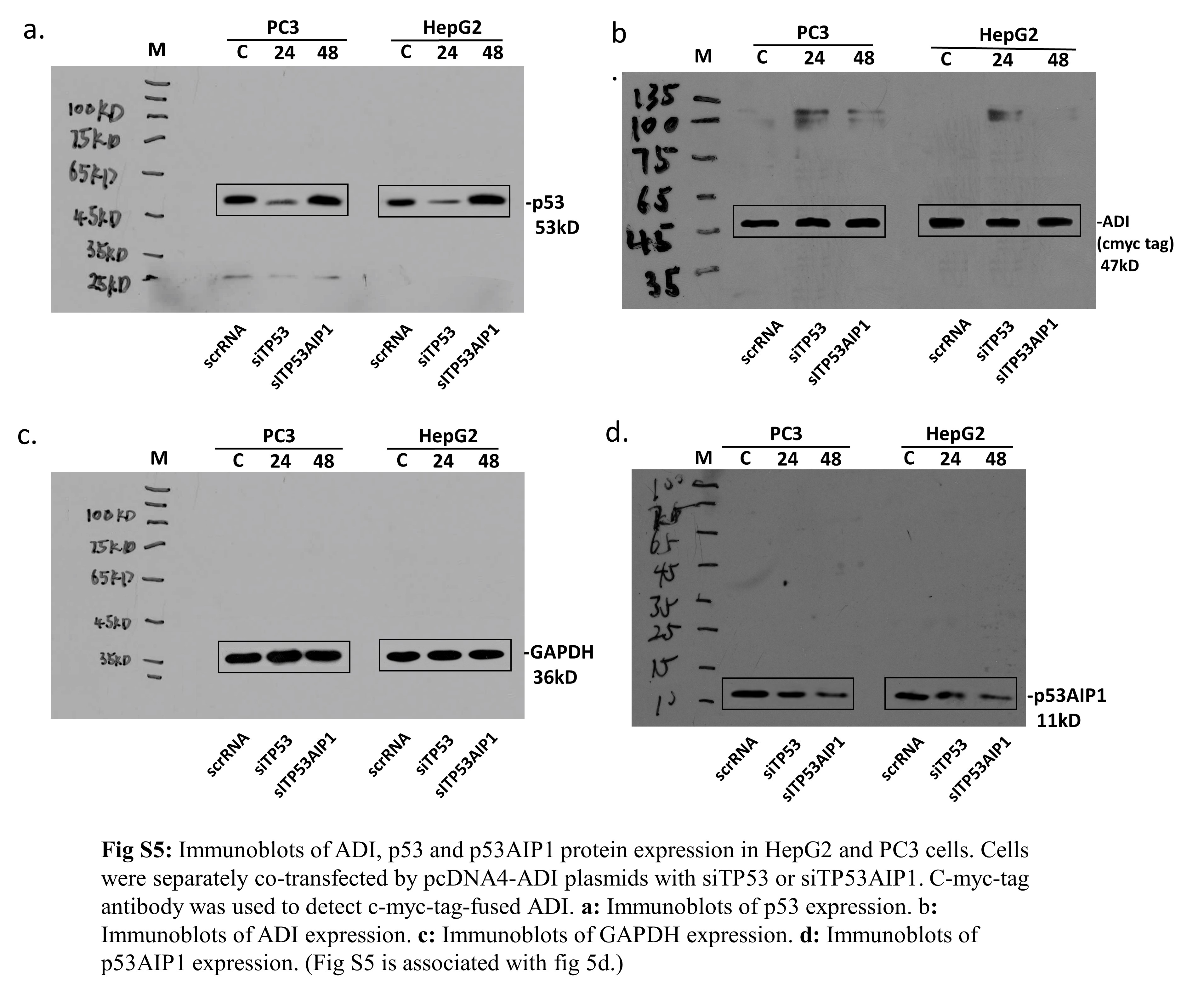

Supplement: Supplementary file 5 — Additional file 5: Figure S5. is associated with Fig. 5d. [file 12885_2020_7133_MOESM5_ESM.jpg]

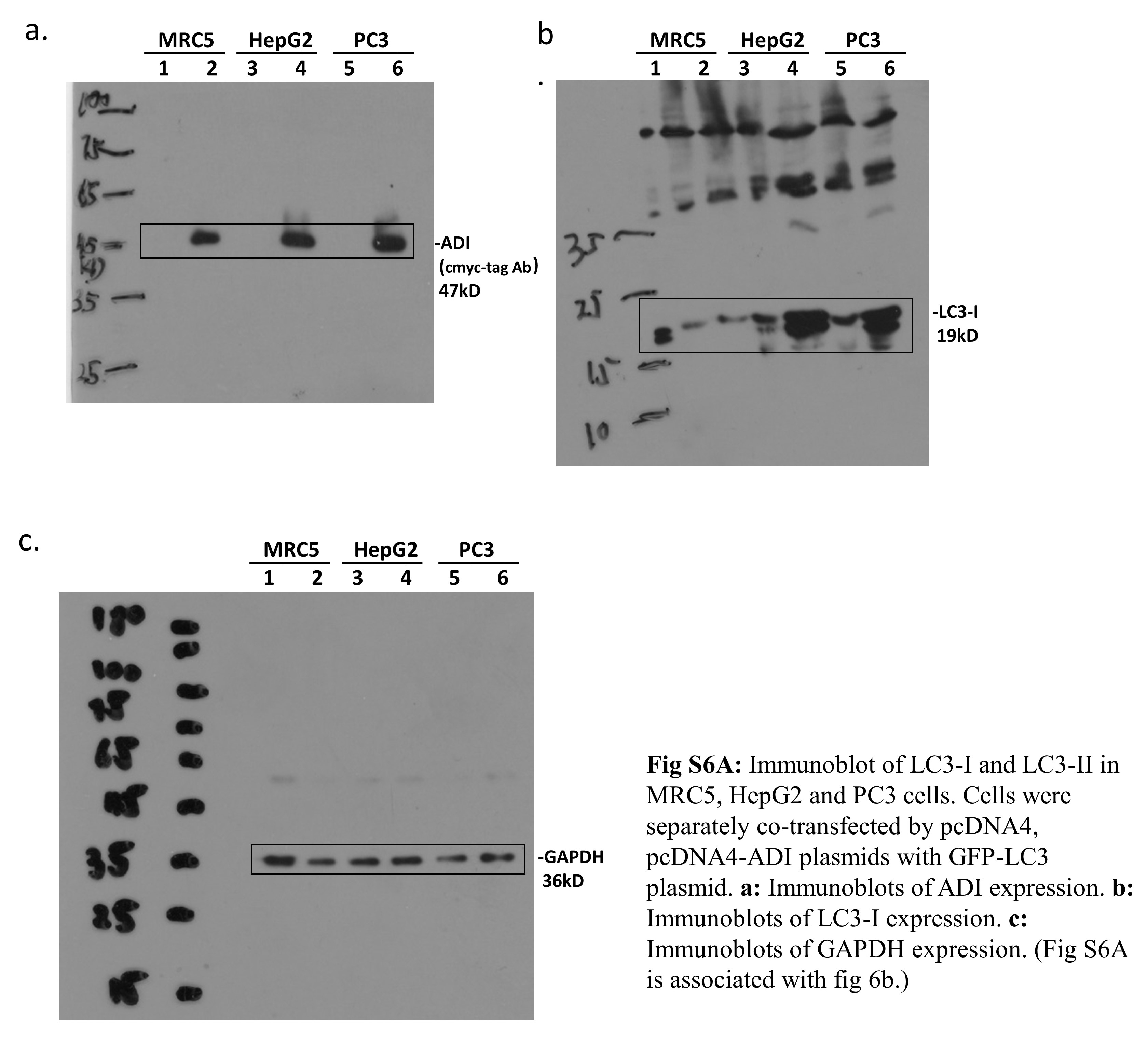

Supplement: Supplementary file 6 — Additional file 6: Figure S6A. is associated with Fig. 6b. [file 12885_2020_7133_MOESM6_ESM.zip › Fig S6AR5.jpg]

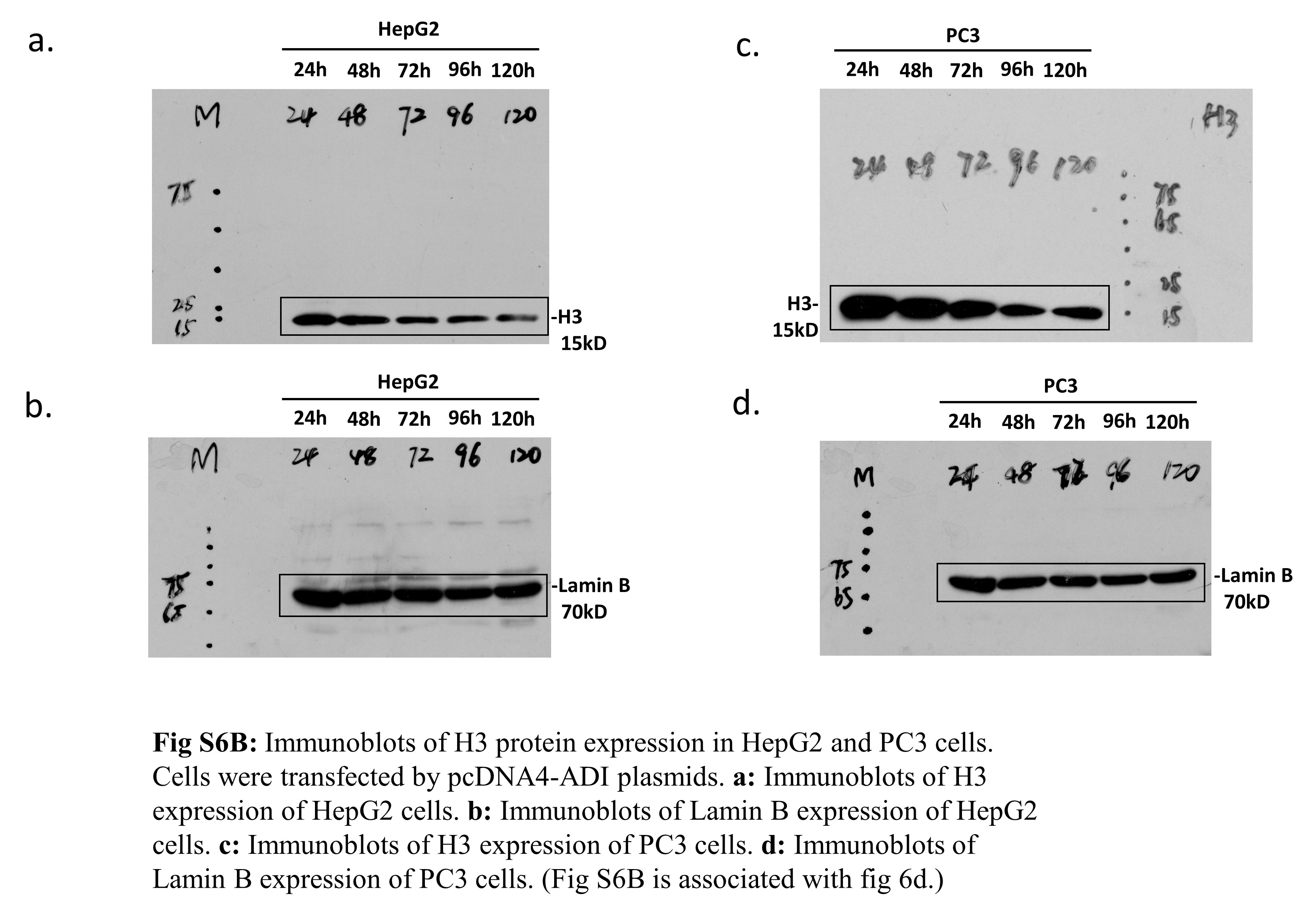

Supplement: Supplementary file 6 — Additional file 6: Figure S6A. is associated with Fig. 6b. [file 12885_2020_7133_MOESM6_ESM.zip › Fig S6BR5.jpg]

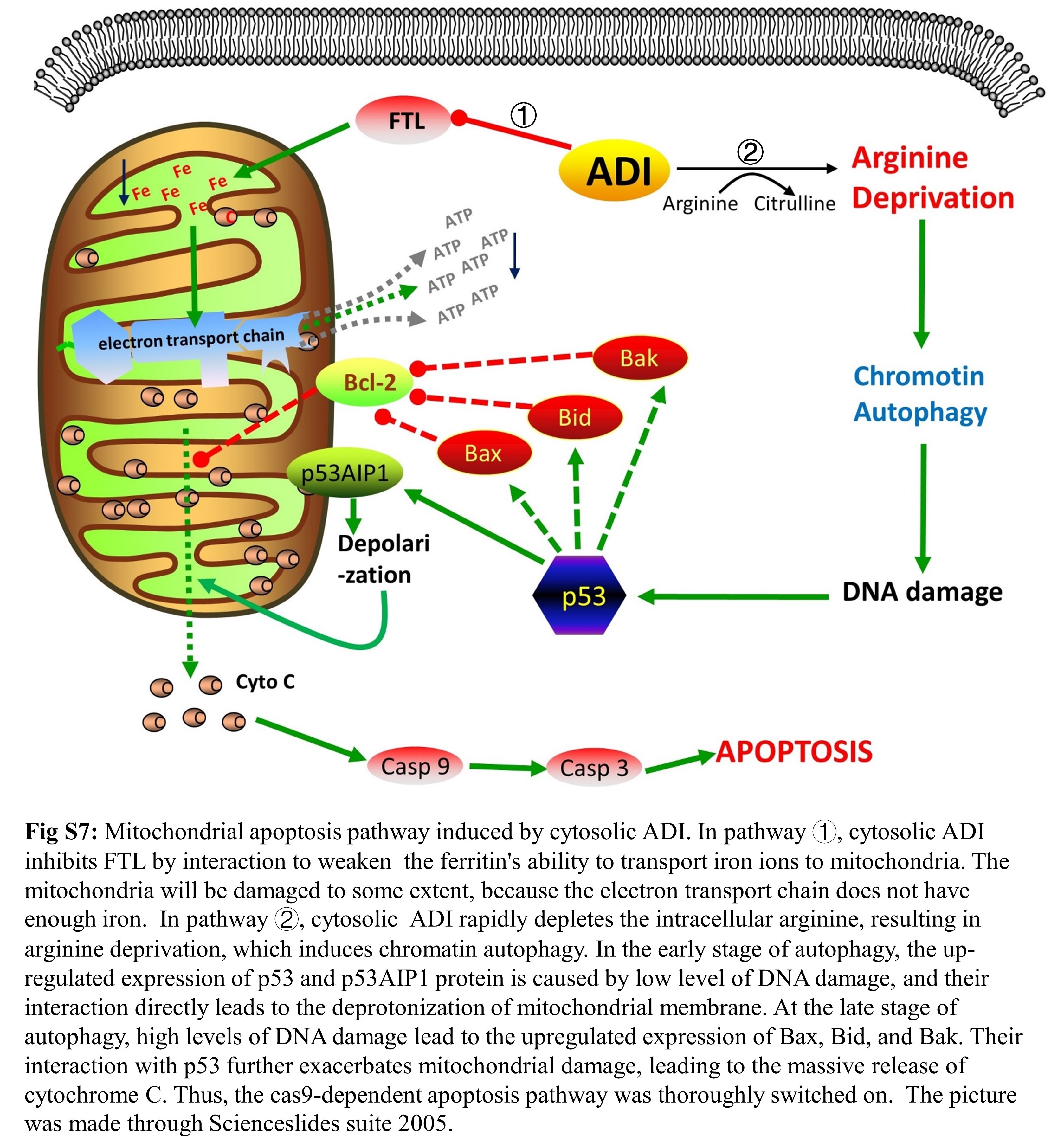

Supplement: Supplementary file 7 — Additional file 7: Figure S6B. is associated with Fig. 6d. Figure S7 is mitochondrial apoptosis pathway induced by cytosolic ADI. [file 12885_2020_7133_MOESM7_ESM.jpg]
