# Supplementary material for: Intracellular expression of arginine deiminase activates the mitochondrial apoptosis pathway by inhibiting cytosolic ferritin and inducing chromatin autophagy
Source: BMC Cancer. 2020 Jul 16;20:665. doi: 10.1186/s12885-020-07133-4 (PMC7367323; doi:10.1186/s12885-020-07133-4)
Supplement: Supplementary file 8 — Additional file 8: Table S1. displays some primers for qPCR experiments. [file 12885_2020_7133_MOESM8_ESM.docx]

**Tab S1, Some primers for qPCR.experiments.**

| **Number** | **Primer Name** | **Sequence (5'to3')** | **Base Number** |
| --- | --- | --- | --- |
| 1 | TP53BP1F | AAGCCAGGCAAGAGAATGAGGC | 22 |
|  | TP53BP1R | GGCTGTTGACTCTGCCTGATTG | 22 |
| 2 | TP53AIP1F | AGACCAGAACCTCTCGGTGATG | 22 |
|  | TP53AIP1R | ACCACGGTGAGAGCAGAGTCTG | 22 |
| 3 | BCL-2F | ATCGCCCTGTGGATGACTGAGT | 22 |
|  | BCL-2R | GCCAGGAGAAATCAAACAGAGGC | 23 |
| 4 | BAXF | TCAGGATGCGTCCACCAAGAAG | 22 |
|  | BAXR | TGTGTCCACGGCGGCAATCATC | 22 |
| 5 | NOXAF | CTGGAAGTCGAGTGTGCTACTC | 22 |
|  | NOXAR | TGAAGGAGTCCCCTCATGCAAG | 22 |
| 6 | BADF | CCAACCTCTGGGCAGCACAGC | 21 |
|  | BADR | TTTGCCGCATCTGCGTTGCTGT | 22 |
| 7 | BIDF | TGGGACACTGTGAACCAGGAGT | 22 |
|  | BIDR | GAGGAAGCCAAACACCAGTAGG | 22 |
| 8 | CASP9F | GTTTGAGGACCTTCGACCAGCT | 22 |
|  | CASP9R | CAACGTACCAGGAGCCACTCTT | 22 |
| 9 | CASP3F | GGAAGCGAATCAATGGACTCTGG | 23 |
|  | CASP3R | GCATCGACATCTGTACCAGACC | 22 |
| 10 | TP53F | CCTCAGCATCTTATCCGAGTGG | 22 |
|  | TP53R | TGGATGGTGGTACAGTCAGAGC | 22 |
| 11 | CYC1F | CTTCGCGGGGTAGTGTTGG | 19 |
|  | CYC1R | GGCCAGACTTCGACGACAA | 19 |
| 12 | FTLF | GTCTCGTCAAGAGGATGGTGGA | 22 |
|  | FTLR | GAAGGATGCCAAAGCTCCACAC | 22 |
| 13 | CYCSF | AAGGGAGGCAAGCACAAGACTG | 22 |
|  | CYCSR | CTCCATCAGTGTATCCTCTCCC | 22 |
| 14 | PUMAF | ACGACCTCAACGCACAGTACGA | 22 |
|  | PUMAR | CCTAATTGGGCTCCATCTCGGG | 22 |
| 15 | BAKF | TTACCGCCATCAGCAGGAACAG | 22 |
|  | BAKR | GGAACTCTGAGTCATAGCGTCG | 22 |
