# Supplementary material for: Intracellular expression of arginine deiminase activates the mitochondrial apoptosis pathway by inhibiting cytosolic ferritin and inducing chromatin autophagy
Source: BMC Cancer. 2020 Jul 16;20:665. doi: 10.1186/s12885-020-07133-4 (PMC7367323; doi:10.1186/s12885-020-07133-4)
Supplement: Supplementary file 9 — Additional file 9. Cell STR gene type test reports and cell purchase certificate [file 12885_2020_7133_MOESM9_ESM.pdf]

STR 基因型检测报告

一、客户信息	
客户姓名：郑忠亮	客户编号：SHJY170801
电话： 18186492975	单位：武汉大学生命科学院
电邮： biochem@whu.edu.cn	湖北省武汉市武昌区珞珈山路 16 号武汉大学生命科学学院 4008 实验室
**二、样品及实验信息**	
样品数量：3	收样日期：2019-08-01
待测位点：22	待测位点总数：66
**三、实验方法**：详见报告第四项。	
检测仪器：ABI9700 型 PCR 仪 ABI3130XL 遗传分析仪	
**四、检测结果：**<br>报告 1 份，包含 3 个样本，每个样本检测 22 个位点。	
引物探针是否返还客户： □是 ■否	样品是否返还客户： □是 ■否

报告声明：

1.本报告只对送检的来样负责。

2.检验报告上的检验结果和检验单位名称，未经同意不得用于广告、评优及商业宣传。

3. 对本报告有异议，请于收到报告之日起十五日内以书面方式提出，逾期不予受理。

4. 对纸质检验报告涂改、增删，或未加盖检验单位印章的复印件均无效。

5.报告发出后，本公司负责保存样品三个月。

报告完成人：王文进	审核： 徐雷	批准：王成
日期：19-08-12	日期：19-08-15	日期：19-08-18

上海基屹生物科技有限公司

一、实验原理：利用荧光标记引物扩增微卫星位点，产物变性后通过毛细管电泳分析，根据 PCR 扩增片段的大小及个数确定基因型。

二、实验目的：对目标样本进行 22 个 STR 位点检测。

三、实验仪器：ABI 9700 PCR 扩增仪，ABI3130XL 遗传分析仪等。

四、实验方案：

1、构建 PCR 反应体系：将 PCR 各组分以下表比例混匀，并震荡离心。

反应组份	加样量（μL）
Reaction Mix	4.0
Primers	2.0
C-Taq 酶	0.4
DNA 模板	1.0
sdH2O	2.6

2、PCR 循环：在 ABI 9700 扩增仪上以下表方式进行参数设置。

温度	时间	循环
95℃	2min	
94℃	30sec	10 个循环
60℃	1min	
72℃	1min	
90℃	30sec	20 个循环
58℃	1min	
72℃	1min	
72℃	10min	

3、PCR 产物变性：取 1ulPCR 产物加入 10μL 配制好的 Hi-Di、SIZ-500 混合液（10:0.3）中，加入 Ladder 对照，平板离心机 3000rpm 离心 3min，95℃变性 5min 后迅速放入冰水浴 3min。

4、电泳：将电泳板放入 ABI3130XL 遗传分析仪，按照设定的电压和进样时间进行电泳。

五、实验结果：实验结果通过 GeneMapper3.2 分析软件进行分析，确定样本分型，结果如下表：

遗传标记	PC-3	HEPG2	MRC5
AMEL	X	XY	XY
D3S1358	16	15/16	15/17
D13S317	11	9/13	11/14
D7S820	8/11	10	10/11
D16S539	11	12/13	9/11
Penta E	10/17	15/20	12/16
D2S441	10/11	11.3/14	10/11
TPOX	8/9	8/9	8
TH01	6/7	9	8
D2S1338	18/20	19/20	20
CSF1PO	11	10/11	11/12
Penta D	9	9/13	12
D10S1248	16	13	13
D19S433	14	15.2	14/15
vWA	17	17	15
D21S11	29/31.2	29/31	31.2
D18S51	14/15	13/14	15/21
D6S1043	18	13	11/19
D8S1179	13	15/16	13
D5S818	13	11/12	11/12
D12S391	21	21/25	20/22
FGA	24	22/25	21/23

(一) 检验情况

样本名称	多等位基因	匹配细胞系	细胞库	EV 值	匹配说明
PC-3	无	465	DSMZ	1	完全匹配
HEP-G2	无	180	DSMZ	1	完全匹配
MRC-5	无	CCL-171	DSMZ	1	完全匹配

(二)样本基因型检验结果

多等位基因指三等位及以上基因现象。

本次检测各细胞分型结果良好。

(三)样本描述

PC-3：该株细胞DNA 分型在细胞系检索中找到完全匹配的细胞系，DSMZ 数据库显示细胞名为PC-3，细胞号对应465。本次检测在该细胞系中没有发现多等位基因。

HEP-G2：该株细胞DNA 分型在细胞系检索中找到完全匹配的细胞系，DSMZ 数据库显示细胞名为HEP-G2，细胞号对应180。本次检测在该细胞系中没有发现多等位基因。

MRC-5：该株细胞DNA 分型在细胞系检索中找到完全匹配的细胞系，DSMZ 数据库显示细胞名为MRC-5，细胞号对应CCL-171。本次检测在该细胞系中没有发现多等位基因。

备注：

STR 数据库比对

本公司采用DSMZ tools 进行细胞系比对，其中包含来自于ATCC, DSMZ, JCRB 和RIKEN 数据库的2455 个细胞系STR 数据。如果待检测细胞未收录于以上细胞库或这是自行建立的新细胞系将无法进行比对，用户需根据细胞分型结果自行与其他数据库进行比对。

附样本分型图谱截图：

PC-3 号样本：

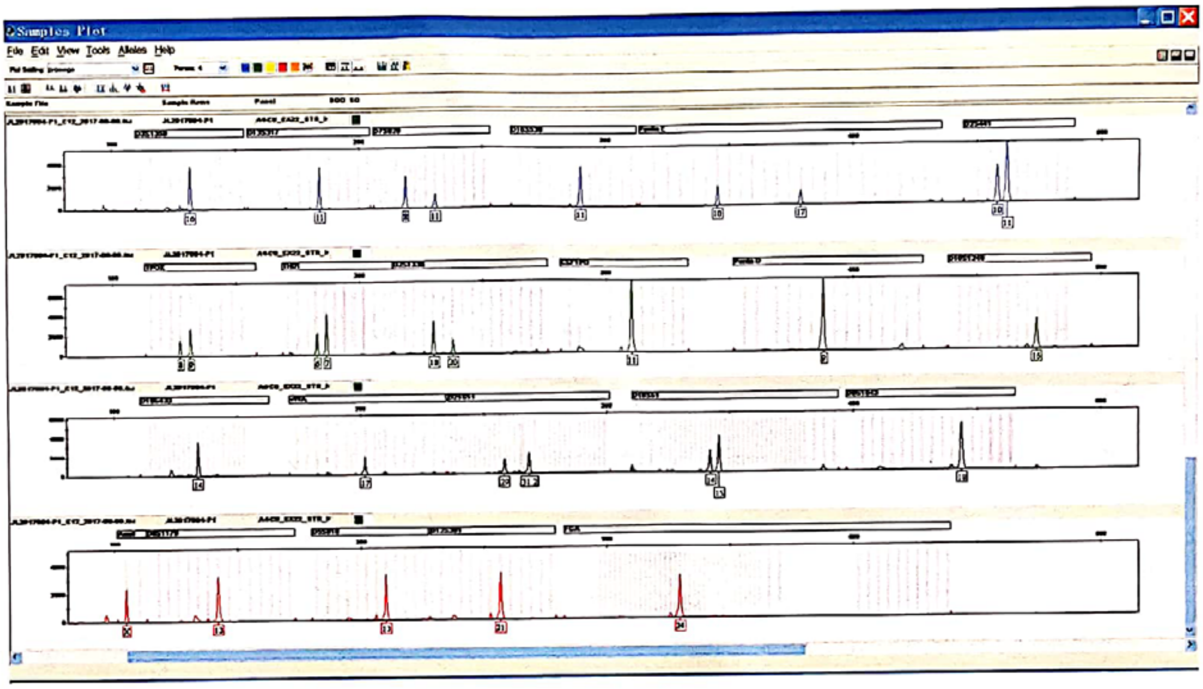

HEP-G2 号样本：

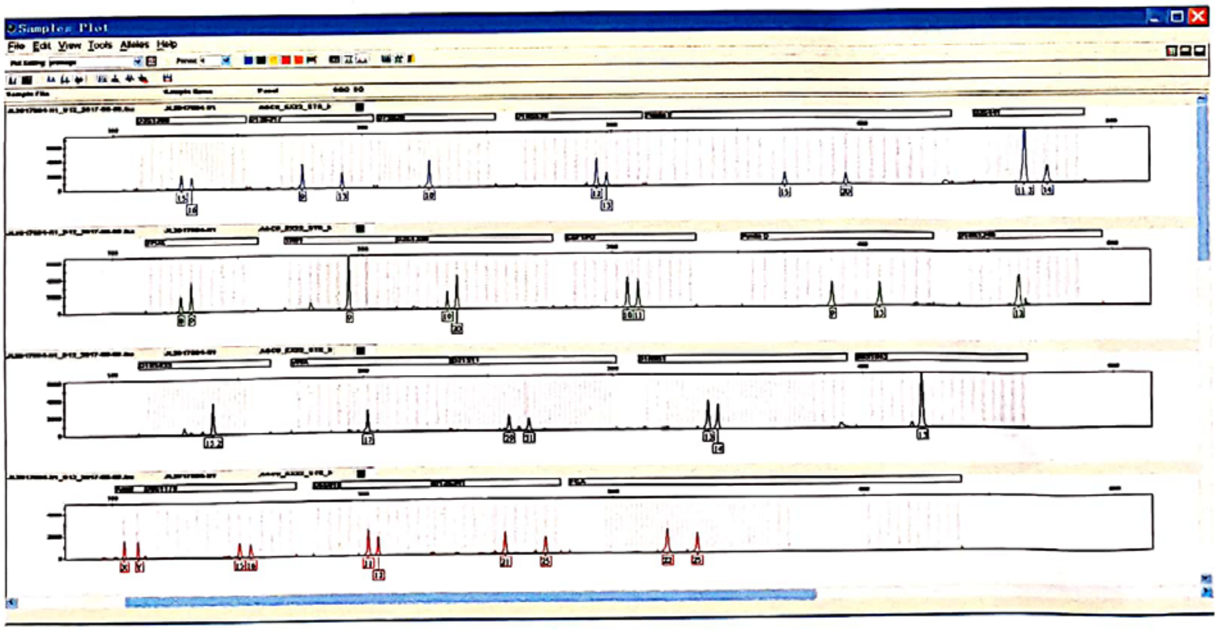

MRC-5 号样本：

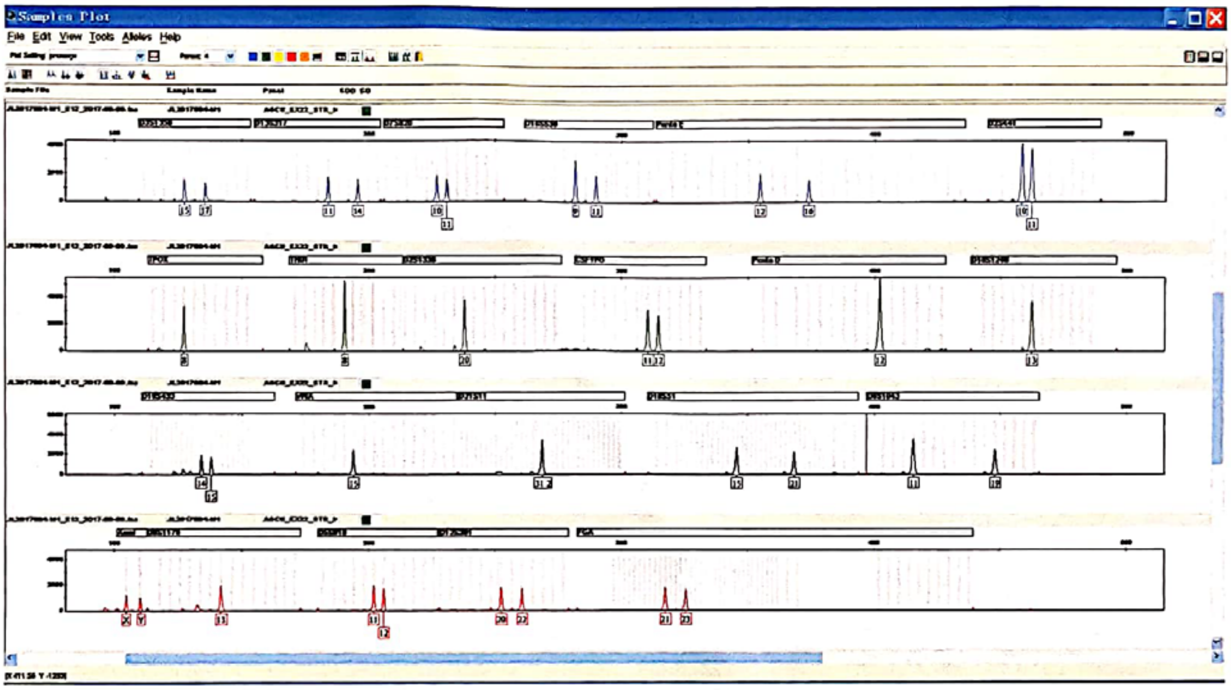

中国典型培养物保藏中心

CHINA CENTER FOR TYPE CULTURE COLLECTION (CCTCC)

Wuhan University, Wuhan 430072, China

Phone: 86-027-68754712 **Fax:** 86-027-68754833 **Email:** cctcccell@whu.edu.cn

兹证明武汉大学生科院郑忠亮老师于 2017 年 7 月 4 日于中国典型培养物保藏中心（CCTCC）购买 HepG2 细胞，此细胞经细菌，真菌和支原体检测后为阴性。细胞株已经过身份认证，无其它细胞污染。我库细胞在所有检测结果确认无误后进行保藏；细胞复苏生长后用 T25 细胞培养瓶罐装提供给使用者。

中国典型培养物保藏中心

2017 年 9 月 11 日

中国典型培养物保藏中心 业务专用章

中国典型培养物保藏中心

CHINA CENTER FOR TYPE CULTURE COLLECTION (CCTCC)

Wuhan University, Wuhan 430072, China

Phone: 86-027-68754712 **Fax:** 86-027-68754833 **Email:** cctcccell@whu.edu.cn

兹证明武汉大学生科院郑忠亮老师于 2017 年 7 月 4 日于中国典型培养物保藏中心（CCTCC）购买 MRC-5 细胞，此细胞经细菌，真菌和支原体检测后为阴性。细胞株已经过身份认证，无其它细胞污染。我库细胞在所有检测结果确认无误后进行保藏；细胞复苏生长后用 T25 细胞培养瓶罐装提供给使用者。

中国典型培养物保藏中心

2017 年 9 月 11 日

中国典型培养物保藏中心

CHINA CENTER FOR TYPE CULTURE COLLECTION (CCTCC)

Wuhan University, Wuhan 430072, China

Phone: 86-027-68754712 **Fax:** 86-027-68754833 **Email:** cctcccell@whu.edu.cn

---

兹证明武汉大学生科院郑忠亮老师于 2017 年 7 月 4 日于中国典型培养物保藏中心（CCTCC）购买 PC-3 细胞，此细胞经细菌，真菌和支原体检测后为阴性。细胞株已经过身份认证，无其它细胞污染。我库细胞在所有检测结果确认无误后进行保藏；细胞复苏生长后用 T25 细胞培养瓶罐装提供给使用者。

中国典型培养物保藏中心

2017 年 9 月 11 日

中国典型培养物保藏中心
业务专用章
